# Genome-Wide Identification and Expression Analyses of Aquaporin Gene Family during Development and Abiotic Stress in Banana

**DOI:** 10.3390/ijms160819728

**Published:** 2015-08-20

**Authors:** Wei Hu, Xiaowan Hou, Chao Huang, Yan Yan, Weiwei Tie, Zehong Ding, Yunxie Wei, Juhua Liu, Hongxia Miao, Zhiwei Lu, Meiying Li, Biyu Xu, Zhiqiang Jin

**Affiliations:** 1 Key Laboratory of Biology and Genetic Resources of Tropical Crops, Institute of Tropical Bioscience and Biotechnology, Chinese Academy of Tropical Agricultural Sciences, Haikou 571101, Hainan, China; E-Mails: huwei2010916@126.com (W.H.); yimiyangguamghxw@163.com (X.H.); yanyan@itbb.org.cn (Y.Y.); tieweiwei@itbb.org.cn (W.T.); dingzehong@itbb.org.cn (Z.D.); weiyunxie1989@126.com (Y.W.); juhua69@126.com (J.L.); miaohongxia@itbb.org.cn (H.M.); luzhiweihn@126.com (Z.L.); limeiying@itbb.org.cn (M.L.); 2 College of Life Science and Technology, Huazhong University of Science & Technology (HUST), Wuhan 430074, Hubei, China; E-Mail: huangchao2005@hust.edu.cn; 3 Key Laboratory of Genetic Improvement of Bananas, Hainan Province, Haikou Experimental Station, Chinese Academy of Tropical Agricultural Sciences, Haikou 570102, Hainan, China

**Keywords:** Aquaporin (AQP), abiotic stress, banana, development, expression analysis, fruit postharvest ripening

## Abstract

Aquaporins (AQPs) function to selectively control the flow of water and other small molecules through biological membranes, playing crucial roles in various biological processes. However, little information is available on the AQP gene family in bananas. In this study, we identified 47 banana AQP genes based on the banana genome sequence. Evolutionary analysis of AQPs from banana, Arabidopsis, poplar, and rice indicated that banana AQPs (MaAQPs) were clustered into four subfamilies. Conserved motif analysis showed that all banana AQPs contained the typical AQP-like or major intrinsic protein (MIP) domain. Gene structure analysis suggested the majority of MaAQPs had two to four introns with a highly specific number and length for each subfamily. Expression analysis of MaAQP genes during fruit development and postharvest ripening showed that some MaAQP genes exhibited high expression levels during these stages, indicating the involvement of MaAQP genes in banana fruit development and ripening. Additionally, some MaAQP genes showed strong induction after stress treatment and therefore, may represent potential candidates for improving banana resistance to abiotic stress. Taken together, this study identified some excellent tissue-specific, fruit development- and ripening-dependent, and abiotic stress-responsive candidate MaAQP genes, which could lay a solid foundation for genetic improvement of banana cultivars.

## 1. Introduction

Aquaporins (AQPs), belonging to the major intrinsic protein (MIP) superfamily, function to selectively control the flow of water and other small molecules, such as CO_2_, glycerol, and boron, through biological membranes [1,2,3,4,5]. AQPs exist in bacteria, fungi, animals, and plants [6]. AQPs of most plant species are typically subdivided into four major groups based on amino acid sequence homology and protein subcellular localization [7], including plasma membrane intrinsic proteins (PIPs) [8], tonoplast intrinsic proteins (TIPs) [9], nodulin 26-like intrinsic proteins (NIPs) [10], and small basic intrinsic proteins (SIPs) [11]. Three additional subfamilies, including GlpF-like intrinsic proteins (GIPs), the hybrid intrinsic proteins (HIPs), and uncategorized members designated X intrinsic proteins (XIPs), have been recently reported in the nonvascular moss *Physcomitrella patens* [12,13]. XIPs are found in protozoa, fungi, and certain land plant species, such as *Populus trichocarpa*, *Nicotiana tabacum*, *Solanum lycopersicon*, and *Solanum tuberosum* [14].

There are highly conserved structures in plant AQPs, in which six membrane-spanning α-helices are linked by five short loops with their N- and C-termini always towards the cytosol [15]. Two of these loops (Loop B and E) contain highly conserved Asp-Pro-Ala (NPA) motifs which play a major role in the formation of water-selective channels [11]. Other important residues in aquaporin sequences are the ones forming the aromatic/arginine selectivity filter (ar/R). This region is formed by four residues towards the extracellular side approximately 8 Å from the NPA region. The four residues are contributed by helix2 (H2), helix 5 (H5), and loop E (LE1 and LE2) [16,17]. The ar/R region has been considered to serve as a selectivity filter for the transport of substrates, determine the transport rate of the protein by acting as a size-exclusion barrier, and provide key hydrogen bonds and van der Waals contacts for the transported solutes and/or water molecules [18].

AQPs effectively mediate fast transmembrane transport of water during plant growth and development processes, such as seed germination, cell elongation, stomatal movement, phloem loading and unloading, and reproductive growth [19,20,21]. Physiological and biochemical evidence has shown that phosphorylation of a putative plasma membrane AQP (PM-AQP) leads to activation of water channels and regulates temperature-dependent opening of tulip petals [20]. Another study demonstrated that expression of *RcPIP2-1* correlated with the elongation activity of etiolated hypocotyls, and the elongating regions of the hypocotyls showed high hydraulic conductivity [19]. Moreover, genetic analysis also revealed the crucial roles of AQPs in plant growth and development processes. GhPIP2 proteins were reported to form hetero-oligomers to modulate their activities in rapid fiber elongation of cotton [21]. Collectively, these studies support that transport of water driven by AQPs is involved in plant growth and development processes.

A large number of studies have also suggested that abiotic stress, including drought, salt, and cold, could induce transcriptional accumulation of *AQPs*, and further genetic analysis has confirmed the function of *AQPs* in plants tolerant to abiotic stress [22,23]. *TaAQP7* and *TaAQP8* expression was shown to be induced after drought and salt treatments, respectively, and overexpression of *TaAQP7* and *TaAQP8* in tobacco was found to result in improved resistance to drought and salt stresses, respectively [22,23]. *MusaPIP1;2*-overexpressing banana plants have shown increased resistance to drought, salt, and cold stresses [15]. Heterologous expression of banana *MaPIP1;1* in *Arabidopsis* was shown to confer tolerance to salt and drought stress by reducing membrane injury, improving ion distribution, and maintaining osmotic balance [24]. Recently, constitutive and stress-inducible overexpression of *MusaPIP2;6* in banana was shown to lead to an increased resistance to salt stress [25]. Taken together, these results demonstrate that AQPs mainly play a positive role in plant responses to abiotic stress.

Genome-wide analyses have identified 35 AQP genes in *Arabidopsis* [7], 34 in rice [26,27], 36 in maize [11], and 53 in Chinese cabbage [5]. However, less information is available for this gene family in banana. Banana is a large annual monocotyledonous herbaceous plant and one of the most important fruit crops. Whole-genome sequencing of banana enables us to perform genome-wide analysis of banana genes. Since AQPs play a crucial role in plant growth, development, and responses to various stresses, this gene family was selected for genome-wide analyses in banana. In the present study, we identified 47 banana AQP (MaAQP) genes and analyzed their phylogenetic relationship, gene structure, protein motifs, and expression patterns during development, ripening, and abiotic stress. The identification and comprehensive investigation of the AQP gene family in banana will provide useful information for future research on genetic improvement of banana quality and resistance to abiotic stress.

## 2. Results

### 2.1. Identification of AQP Genes in Banana

**Figure 1:**
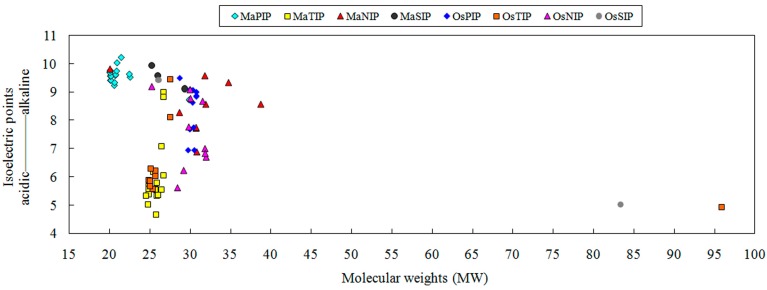
Putative isoelectric point and relative molecular weight of plasma membrane intrinsic proteins (PIPs), tonoplast intrinsic proteins (TIPs), nodulin 26-like intrinsic proteins (NIPs) and small basic intrinsic proteins (SIPs) from banana and rice with ExPASy proteomics server database. MaPIP, MaTIP, MaNIP and MaSIP indicate AQPs from banana. OsPIP, OsTIP, OsNIP and OsSIP indicate AQPs from rice.

To identify AQP family members from banana, a genome-wide search was carried out using both Hidden Markov Model and BLAST searches with 35 AQP genes from *Arabidopsis* and 34 AQP genes from rice as queries. A total of 47 non-redundant MaAQP genes were identified from the banana genome. All identified sequences of amino acids and full length cDNAs for banana AQPs were listed in Tables S1 and S2, respectively. Conserved domain and multiple sequence alignment analyses suggested that all identified banana AQPs contained the typical AQP family MIP domain. Prediction of transmembrane domains (TMDs) showed that most MaAQPs (26 of 47%, 55%) contained six TMDs, whereas the other 21 MaAQPs had 4, 5 or 7 predicted TMDs (Table S3). Further, we identified the NPA motifs and ar/R selectivity filter sequences of banana AQPs by multiple sequence alignment analysis with tomato AQPs as references (Table S4, Figures S1–S5) [28]. Both in the MaPIP and MaTIP subfamilies, the two NPA motifs are conserved with typical Asp-Pro-Ala residues. In the MaNIP subfamily, all the MaNIPs also show typical Asp-Pro-Ala residues, except for MaNIP3-2, where the alanine is replaced by a serine residue in the first NPA and by a valine in the second NPA. In the MaSIP subfamily, the first NPA have extensive variation, among which the alanine is replaced by threonine for MaSIP1-1 or by leucine for MaSIP2-1 and MaSIP2-2, whereas the second NPA are completely conserved. The ar/R filter showed an increased subfamily-specific sequence in comparison to the two NPA motifs. In the MaPIP subfamily, all MaPIPs showed an ar/R filter configuration typical for conserved residues in other species (F, H, T, R). In the MaTIP subfamily, the ar/R is formed by H/Q in H2, I/S/V/T in H5, A/G in LE1 and V/R in LE2. The MaNIP subfamily showed W/G/A in H2, V/S/I in H5, A/G in LE1 and R in LE2. In the MaSIP subfamily, the ar/R is constituted by L/Y/F in H2, T/K in H5, P/G in LE1 and N/S in LE2. To determine their protein characteristics, the isoelectric point (pI) and relative molecular mass (RMW) of banana AQP amino acid sequences were calculated using ExPASY (Table S5). The 47 predicted AQP proteins from banana ranged from 188 to 359 amino acid residues in length, with RMWs varying from 20.0 to 38.7 KDa. The protein characteristics of AQPs from banana and rice were compared according to their pI and RMW (Figure 1). In the PIP subfamily, the pI of most MaPIPs ranged from 9 to 10, and the RMW was approximately 20 kDa, whereas the pI of most OsPIPs ranged from 7 to 10 with a RMW of approximately 30 kDa. In the TIP subfamily, the pI of most TIPs was between 5 and 7 and the RMW was approximately 25 kDa both in banana and rice. In the NIP subfamily, the MaNIPs showed a broad RMW, whereas the OsNIPs displayed a wide range of pI. In the SIP subfamily, most SIPs from banana and rice showed similar RMW and pI. Generally, the protein characteristics of PIP and NIP subfamilies in banana were different from that in rice, implying their functional diversity.

### 2.2. Phylogenetic Analysis of AQP Proteins

**Figure 2:**
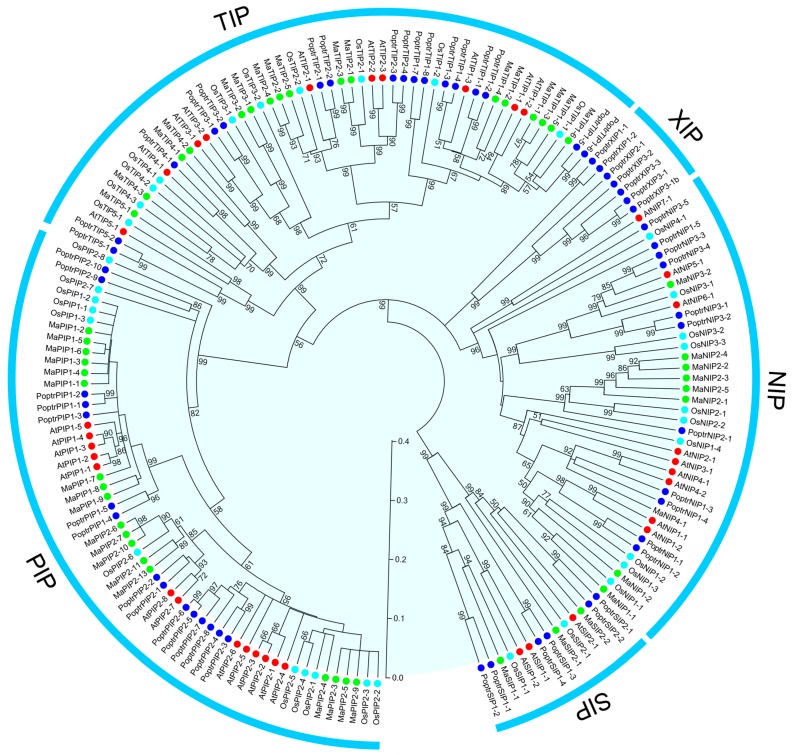
Phylogenetic tree of Aquaporins (AQPs) from banana, *Arabidopsis*, poplar and rice. A neighbor-joining tree was constructed using ClustalX and MEGA5.0 software with 1000 bootstraps. Five subfamilies were indicated as PIP, SIP, NIP, TIP and X intrinsic proteins (XIPs). Green round, 47 AQP proteins from banana; red round, 35 AQP proteins from *Arabidopsis*; light blue, 33 AQP proteins from rice; dark blue, 56 AQP proteins from poplar.

To study the evolutionary relationships between AQP proteins from banana, *Arabidopsis*, poplar and rice, a neighbor-joining phylogenetic tree was created based on the alignments of their amino acid sequences (Figure 2, Table S6). The results showed that all identified AQPs from banana were clearly divided into four different subfamilies (PIP, TIP, NIP, and SIP), together with AQPs from *Arabidopsis*, poplar and rice. No banana AQP members were assigned into the XIP subfamily branched by poplar AQPs. According to the phylogenetic relationship, 18 MaAQPs were classified as PIPs, 17 as TIPs, 9 as NIPs, and 3 as SIPs. Furthermore, the PIPs, TIPs, NIPs, and SIPs were subdivided into two, five, four, and two subgroups (PIP1 and PIP2; TIP1, TIP2, TIP3, TIP4, and TIP5; NIP1, NIPP2, NIP3, and NIP4; SIP1 and SIP2), respectively. Overall, AQPs from banana had a closer relationship with those from rice than those from *Arabidopsis* and poplar, which is in line with current opinions in plant evolution. Moreover, BLASTP analysis further supports the phylogenetic classification of MaAQPs with each subfamily showing high identity with AQPs from other species (Table S6).

### 2.3. Gene Structure of AQP Genes in Banana

**Figure 3:**
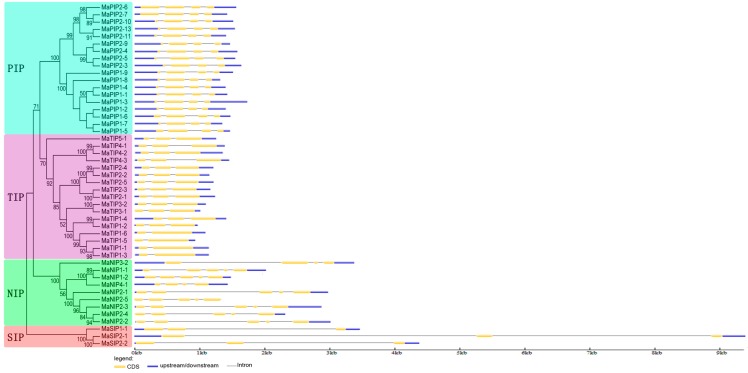
Exon-intron structure analyses of banana *AQPs*. Exon-intron structure analyses were conducted using the GSDS database. Lengths of exons and introns of each *MaAQP* gene are displayed proportionally.

Exon-intron structural diversity often plays a key role in the evolution of gene families and can provide additional evidence to support phylogenetic groupings [29,30]. The exon-intron structure of MaAQP genes was detected according to their evolutionary classification. As shown in Figure 3, divergence of the AQP family is consistent with classification results presented in Figure 2. All *MaPIP* genes had three introns. Thirteen of the 17 *MaTIP* genes had two introns, while the remaining four *MaTIPs* contained only one intron. The number of introns in *MaNIPs* ranged from three to four. *MaNIP1-1*, *MaNIP1-2*, and *MaNIP2-1*, *-2*, *-3*, *-4*, and *-5* had four introns; and *MaNIP3-2* and *MaNIP4-1* contained three. All *MaSIP* genes contained two long introns. Within this frame, paralogous gene pairs generally shared highly similar exon-intron structures, such as *MaPIP2-6* and *MaPIP2-7*, *MaPIP1-3* and *MaPIP1-4*, and *MaTIP1-1* and *MaTIP1-3*. On the whole, the number and length of exons and introns was highly specific for each subfamily (Figure 3). The divergent gene structures among the different phylogenetic subgroups suggest that genes evolved into diverse exon-intron structures to accomplish different functions in the banana genome.

### 2.4. Conserved Motifs of AQP Proteins in Banana

**Figure 4:**
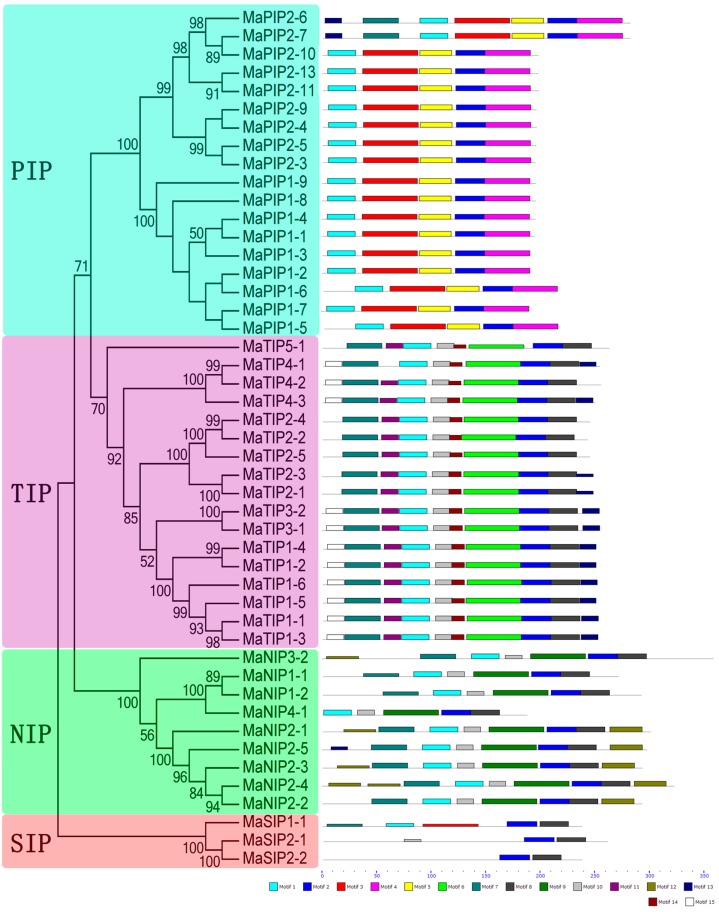
Motif analyses of banana AQPs. All motifs were identified by the MEME database with complete amino acid sequences of MaAQPs. Lengths of motifs for each MaAQP protein are displayed proportionally.

To detect the structural diversity and predict the function of MaAQP proteins, a total of 15 conserved motifs in banana AQPs were found by MEME software and further annotated by InterPro Scan 5 (Figure 4, Table S7). The results showed that nine (1–9) out of 15 motifs were annotated as AQP-like or MIP domains, which are basic characteristics of the AQP family. All MaAQP proteins identified contained these conserved domains. All AQPs in the PIP subfamily contained motifs 1–5. Moreover, closely related MaPIP2-6 and MaPIP2-7 also had motifs 7 and 13, in addition to motifs 1–5. In the TIP subfamily, all members contained motifs 1, 2, 6–8, 10, and 14. In the SIP subfamily, all three members contained motifs 2 and 8. In the NIP subfamily, all members had motifs 1, 2, and 8–10. Thus, each subfamily of banana AQP shared the same conserved motifs, which supports the phylogenetic classification of the banana AQP family.

### 2.5. Expression Analysis of MaAQP Genes in Different Tissues of Two Banana Varieties

**Figure 5:**
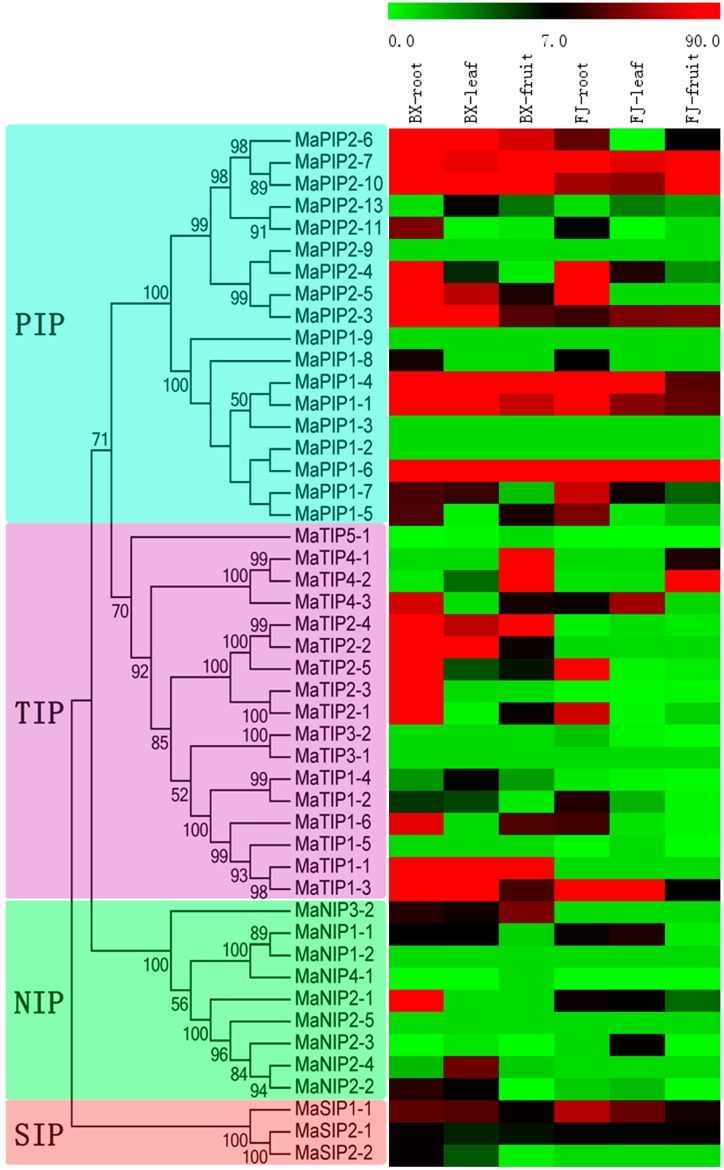
Expression profiles of MaAQP genes in roots, leaves, and fruits of two cultivated banana varieties. The heat-map was established based on MeV software. The scale represents signal intensity of FPKM values.

To provide some clues on the roles of MaAQP genes in banana growth and development, expression profiles of AQP genes in different organs, including roots, leaves, and fruits, were tested in two cultivated banana varieties, BaXi Jiao (*Musa acuminate* L. AAA group cv. Cavendish; hereafter referred to as BX) and Fen Jiao (*Musa* ABB Pisang Awak; hereafter referred to as FJ). The transcripts of 40 MaAQP genes (except for MaPIP2-9, MaPIP1-9, MaPIP1-3, MaPIP1-2, MaTIP3-1, MaNIP1-2 and MaNIP2-5) were captured based on transcriptomic data from each organ, and then a heat-map with phylogenetic analysis was produced to show the expression patterns of MaAQP genes in different organs (Figure 5, Table S8).

In BX, 92.5% (37/40), 85% (34/40), and 82.5% (33/40) of MaAQP genes were expressed in roots, leaves, and fruits, respectively, with 78.4% (29/37), 52.9% (18/34), and 66.7% (20/33) of MaAQP genes showing high expression levels (value > 7) in roots, leaves, and fruits, respectively. Additionally, 80% (32/40) of MaAQP genes were expressed in all organs examined, among which 43.75% (14/32) showed high expression levels (value > 7) in all three organs. In FJ, MaAQP genes with transcription accumulation accounted for 82.5% (33/40), 77.5% (31/40), and 80% (32/40) in roots, leaves, and fruits, respectively, with 66.67% (22/33), 38.71% (12/31), and 34.38% (11/32) showing high expression levels (value > 7) in roots, leaves, and fruits, respectively. Additionally, 72.5% (29/40) of MaAQP genes were expressed in all organs examined, with 24.14% (7/29) showing high expression levels (value > 7) in all three organs.

Comparison of MaAQP gene expression patterns in the different organs of the two banana varieties showed there were more MaAQP genes with high expression levels (value > 7) in all organs of BX than FJ, implying MaAQP genes have a greater contribution to organ development in BX plants. Notably, *MaTIP1-1* and *MaNIP3-2* showed high expression levels (value > 13) in all three organs of BX, whereas there was no transcription accumulation in FJ. Accordingly, *MaTIP2-4* also had extremely high expression levels (value > 59) in all three organs of BX, but low transcription accumulation (value < 1) in FJ. This suggests the crucial roles of these genes in BX organ development. It is important to note that 55% (22/40) of MaAQP genes were expressed in all three organs of BX and FJ, and *MaPIP2-10*, *MaPIP1-1*, *MaPIP2-3*, *MaPIP1-4*, *MaPIP2-7*, and *MaSIP1-1* showed high expression levels (value > 7) in all three organs of BX and FJ.

Additionally, some closely related MaAQP genes showed similar expression patterns in different tissues, among which MaPIP2-7:MaPIP2-10 and MaPIP1-1:PIP1-4 had high expression levels, while MaPIP2-13:MaPIP2-11, MaTIP1-4:MaTIP1-2 and MaSIP2-1:MaSIP2-2 exhibited low expression levels. Conversely, some closely related MaAQP genes showed differentially expressed patterns in different tissues, including MaPIP2-5:MaPIP2-3 and MaTIP1-1:MaTIP1-3.

### 2.6. Expression Analysis of MaAQP Genes in Different Stages of Fruit Development and Ripening of Two Banana Varieties

**Figure 6:**
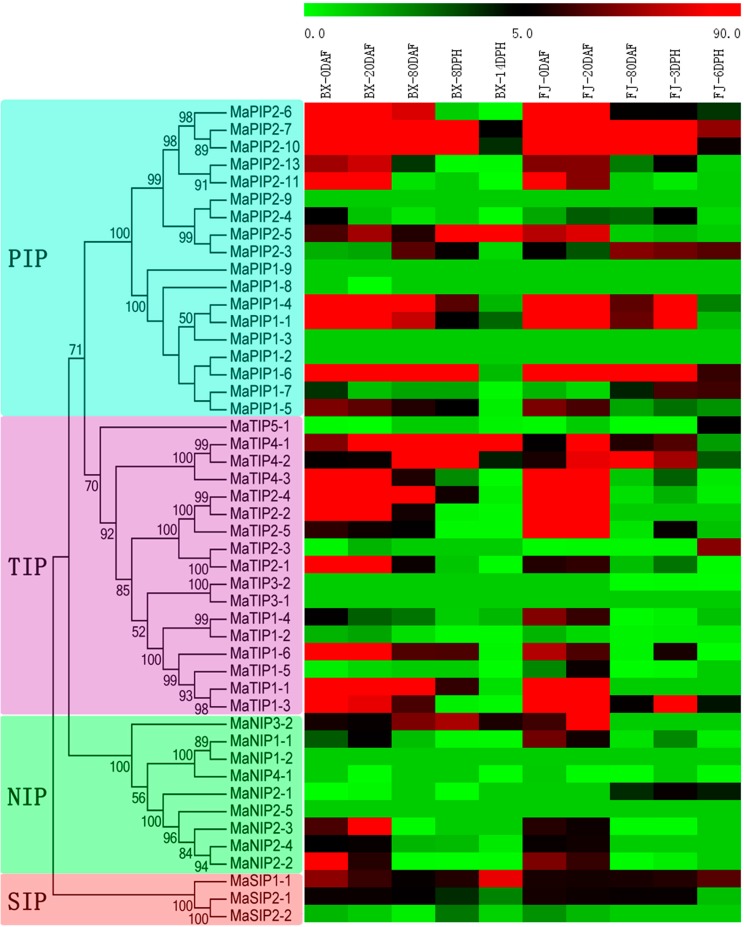
Expression profiles of MaAQP genes at different stages of fruit development and ripening of two cultivated banana varieties. The heat-map was established based on MeV software. The scale represents signal intensity of FPKM values.

To investigate the possible roles of MaAQP genes in fruit development and ripening, transcriptional changes of MaAQP genes were examined at different stages of fruit development and ripening in BX and FJ. Fruit at 0, 20, and 80 DAF in BX and FJ, 8 and 14 DPH in BX, and 3 and 6 DPH in FJ were collected to determine the expression profiles of MaAQP genes. Transcriptome analyses obtained expression data from 40 MaAQP genes (except for MaPIP2-9, MaPIP1-9, MaPIP1-3, MaPIP1-2, MaTIP3-1, MaNIP1-2 and MaNIP2-5) in different stages of fruit development and ripening (Figure 6, Table S9).

In BX, 92.5% (37/40), 97.5% (39/40), 82.5% (33/40), 77.5% (31/40), and 87.5% (35/40) of MaAQP genes showed expression at 0, 20, and 80 DAF, as well as 8 and 14 DPH, respectively. Moreover, 64.87% (24/37), 61.54% (24/39), 57.58% (19/33), 38.71% (12/31) and 11.43% (4/35) of MaAQP genes exhibited high expression levels (value > 10) at 0, 20, and 80 DAF, as well as 8 and 14 DPH, respectively. In addition, 75% (30/40) of MaAQP genes were expressed at all tested fruit stages, with *MaPIP2-5* and *MaTIP4-1* having high expression levels (value > 10) during all stages of fruit development and ripening.

In FJ, transcriptional accumulation accounted for 92.5% (37/40), 90% (36/40), 75% (30/40), 82.5% (33/40), and 72.5% (29/40) of MaAQP genes 0, 20, and 80 DAF, as well as 3 and 6 DPH, respectively. Moreover, 72.97% (27/37), 80.56% (29/36), 33.33% (10/30), 36.36% (12/33), and 24.14% (7/29) of MaAQP genes had high transcriptional accumulation (value > 10) at 0, 20, and 80 DAF, as well as 3 and 6 DPH, respectively. Additionally, 62.5% (25/40) of MaAQP genes were expressed at all tested stages, with *MaPIP2-10*, *MaPIP1-6*, *MaPIP2-7*, and *MaSIP1-1* having high expression levels (value > 10) during all stages of fruit development and ripening.

Generally, similar expression patterns were observed in the examined stages of fruit development and ripening in BX and FJ. The number of MaAQP genes with high expression levels (value > 10) was significantly greater at 0 and 20 DAF than that at the remaining stages in both BX and FJ. In BX and FJ, 55% (22/40) of MaAQP genes were expressed at all examined stages. In contrast, some MaAQP genes showed low or no expression at all stages of fruit development and ripening in BX and FJ, including *MaPIP1-8*, *MaNIP4-1*, *MaTIP3-2*, *MaTIP5-1*, *MaTIP1-2*, and *MaSIP2-2*.

Additionally, some closely related MaAQP genes displayed similar expression patterns during fruit development and ripening, including MaPIP2-7:MaPIP2-10, MaPIP2-13:MaPIP2-11, MaPIP2-9:MaPIP2-4, MaPIP1-4:MaPIP1-1 and MaTIP2-4:MaTIP2-2. In contrast, some closely related MaAQP genes showed different expression patterns, such as, MaPIP2-5:MaPIP2-3, MaPIP1-7:MaPIP1-5, MaTIP4-1:MaTIP4-2 and MaTIP2-3:MaTIP2-1.

### 2.7. Expression Analysis of MaAQP Genes in Response to Cold, Salt, and Osmotic Stresses in Two Banana Varieties

**Figure 7:**
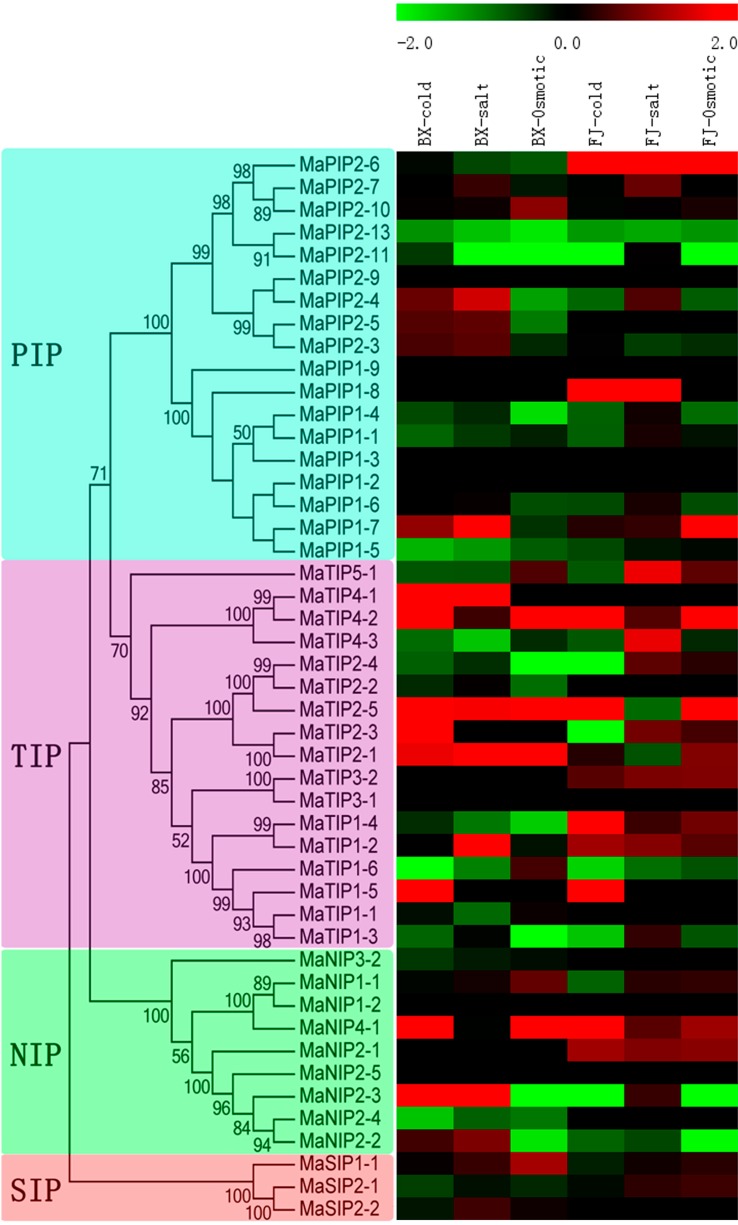
Expression profiles of MaAQP genes in leaves of two cultivated banana varieties responding to cold, salt, and osmotic stresses. Log_2_-based FPKM values were used to create the heat-map with MeV software. The scale represents relative signal intensity of FPKM values.

Accumulated evidence has demonstrated that AQP genes play an important role in conferring plants resistance to abiotic stress, including drought, cold, and salt [3,22,23,24]. Thus, there is a need to investigate the transcriptional response of MaAQP genes to these abiotic stresses, which will benefit further functional characterization of AQP genes under stress conditions in banana. In the present study, a heat-map representing expression profiles of 40 MaAQP genes (except for MaPIP2-9, MaPIP1-9, MaPIP1-3, MaPIP1-2, MaTIP3-1, MaNIP1-2 and MaNIP2-5) was produced using transcriptomic data (Figure 7, Tables S10 and S11).

In BX, transcripts of 45% (18/40), 45% (18/40), and 27.5% (11/40) of MaAQP genes increased after cold, salt, and osmotic treatments, respectively, whereas 47.5% (19/40), 42.5% (17/40), and 57.5% (23/40) decreased after these stress treatments, respectively. Significant changes (*p* < 0.05) of 22.5% (9/40), 22.5% (9/40), and 15% (6/40) of MaAQP genes was found after cold, salt, and osmotic stress, respectively. Notably, 3 (MaTIP2-3, MaTIP1-5, and MaTIP4-2), 7 (MaPIP2-3, MaPIP2-7, MaTIP1-2, MaTIP2-1, MaNIP2-2, MaSIP1-1 and MaSIP2-2) and 1 (MaSIP1-1) genes were significantly induced (*p* < 0.05) after cold, salt, and osmotic treatments, respectively.

In FJ, 30% (12/40), 62.5% (25/40), and 40% (16/40) of MaAQP genes were induced after cold, salt, and osmotic treatments, respectively. In contrast, 52.5% (21/40), 17.5% (7/40), and 37.5% (15/40) were downregulated after cold, salt, and osmotic stress, respectively. Significant changes (*p* < 0.05) of 22.5% (9/40), 7.5% (3/40), and 12.5% (5/40) of MaAQP genes was found after cold, salt, and osmotic stress, respectively. Four (MaPIP2-6, MaTIP1-2, MaTIP1-4 and MaTIP4-2), 2 (MaTIP1-2 and MaTIP4-3) and 2 (MaPIP1-7 and MaNIP2-1) genes showed significant upregulation (*p* < 0.05) under cold, salt, and osmotic stress, respectively.

From above expression data, we can observe that the number of upregulated MaAQP genes by cold stress were more in BX than that in FJ, whereas less in BX than in FJ after salt and osmotic treatments. Notably, *MaPIP2-6* and *MaTIP1-4* were upregulated in FJ with the three treatments, but downregulated in BX. *MaNIP2-1* and *MaTIP3-2* expression was induced in FJ with the three treatments, whereas no transcriptional accumulation was detectable for these two genes in BX. Moreover, *MaTIP4-2* was upregulated in response to cold, salt, and osmotic stress in both BX and FJ, whereas *MaPIP2-13* and *MaPIP1-5* were downregulated.

Additionally, some closely related MaAQP genes displayed similar expression profiles after various stress treatments, such as MaPIP2-13:MaPIP2-11 and MaPIP1-1:MaPIP1-4, Conversely, many closely related MaAQP genes showed different expression patterns, including MaPIP2-7:MaPIP2-10, MaPIP1-7:MaPIP1-5, MaTIP4-1:MaTIP4-2, MaTIP2-4:MaTIP2-2, MaTIP2-3:MaTIP2-1, MaTIP3-1:MaTIP3-2, MaTIP1-4:MaTIP1-2, MaNIP2-4:MaNIP2-2 and MaSIP2-1:MaSIP2-2.

### 2.8. Validation of the Differentially Expressed AQP Genes by qRT-PCR Analysis

**Figure 8:**
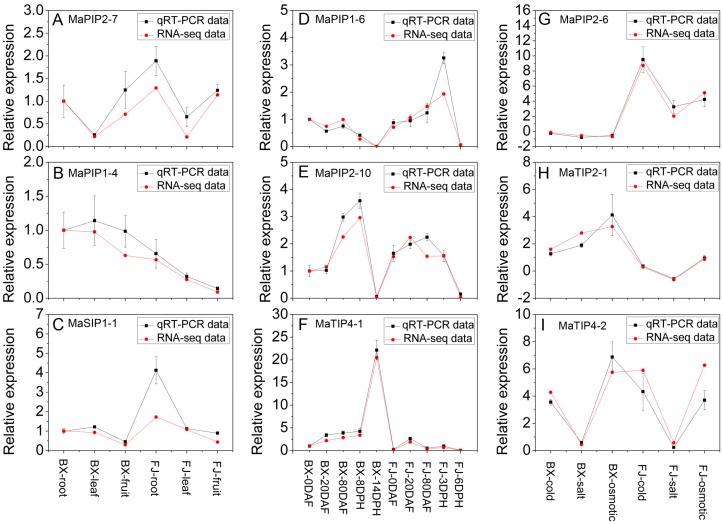
Relative mRNA levels of 9 MaAQP genes in BX and FJ were determined by qRT-PCR analysis. (**A**–**C**) expression patterns of MaPIP2-7, MaPIP1-4 and MaSIP1-1 in different tissues of BX and FJ. The mRNA fold difference was relative to that of BX-root samples used as calibrator; (**D**–**F**) expression patterns of MaPIP1-6, MaPIP2-10 and MaTIP4-1 in different stages of fruit development and ripening in BX and FJ. The mRNA fold difference was relative to that of BX-0DAF samples used as calibrator; (**G**–**I**) expression patterns of MaPIP2-6, MaTIP2-1 and MaTIP4-2 in response to cold, salt and osmotic stresses in BX and FJ. The mRNA fold difference was relative to that of untreated samples used as calibrator. Log_2_-based values were used to display differential expression results. Data are means ± SD of *n* = 3 biological replicates.

According to the RNA-seq data, *MaPIP2-7*, *MaPIP1-4* and *MaSIP1-1* showed high expression levels (value > 7) in all three organs (root, leaf, and fruit) of BX and FJ, MaPIP1-6, MaPIP2-10 and MaTIP4-1 had abundant transcripts (value > 10) during all stages of fruit development and ripening in BX and/or FJ, and MaPIP2-6, MaTIP2-1 and MaTIP4-2 were upregulated by cold, salt and osmotic stresses in BX and/or FJ. These nine differentially expressed MaAQP genes identified in this study were selected for qRT-PCR analysis to validate the RNA-seq data. After normalization, we found that the majority of selected MaAQP genes, except for *MaPIP1-4* in leaf of BX and *MaPIP2-10* in 80 DAF of FJ, showed the same trend and consistent results between RNA-seq data and qRT-PCR data (Figure 8). These results indicate that RNA-seq data are suitable for supplying the expression patterns of AQP genes in two banana varieties.

## 3. Discussion

Bananas, one of the most popular fresh fruits enjoyed worldwide, ranks as the second largest fruit crop and is one of the top five food commodities of the world [15]. However, studies on banana have proceeded at a slow pace in comparison to other important food crops [15]. Banana fruit quality is determined by a series of developmental and ripening processes. Moreover, banana plants are frequently destroyed by various biotic and abiotic stresses during growth and development. However, mechanisms involved in banana fruit development, ripening, and responses to environmental stresses are less known. Because AQPs play important roles in fast transmembrane transport of water during plant growth, development, and stress, they are considered crucial proteins in plants. In the present study, we focused on genome-wide identification and molecular characterization of AQPs during development, ripening, and abiotic stress in banana, which will provide new clues for further functional characterization of AQP genes and genetic improvement of bananas.

### 3.1. Identification and Evolutionary Analysis of MaAQPs

Both HMM and BLAST searches were employed to identify the banana AQP members according to the genome sequence of DH-Pahang (*Musa acuminate*, A-genome, 2*n* = 22). Forty-seven MaAQP genes were identified from the banana genome, indicating the expansion of AQP genes in banana compared to *Arabidopsis* (35) and rice (34) [7,26,27]. This high number of AQPs could be interpreted as a possible functional redundancy of this gene family in banana. Phylogenetic analysis indicated that banana AQPs could be clustered into four different subfamilies (Figure 2), which is consistent with previous reports of AQPs from rice, *Arabidopsis*, maize, and cabbage [5,7,11,27]. Generally, AQPs from banana had a closer relationship with those from rice than those from *Arabidopsis* and poplar, which is in line with current opinions in plant evolution. Gene structure analysis suggested that the number and length of exons and introns was highly specific for each subfamily (Figure 3). The number of MaAQP exon-introns in each subfamily is consistent with that in the common bean and potato [31,32]. Conserved motif analysis showed that each subfamily of banana AQP shared the same conserved motifs (Figure 4). Therefore, conserved motif and gene structure analyses further support the identification and phylogenetic classification of *MaAQPs*.

All MaPIPs contain two typical NPA motifs and highly conserved sequences of ar/R selectivity filter (F, H, T, R) (Table S4, Figures S1 and S2). The same ar/R selectivity filter sequences are also observed in the PIP sub-family of other species, such as, Arabidopsis, maize, tomato and poplar [7,11,23,28]. Accumulated evidences have confirmed that plant PIPs play an important role in water absorption of roots and maintaining leaf hydraulics [22,23,24,33]. In addition to water transport, some studies also reported that PIPs could facilitate the diffusion of CO_2_ in mesophyll and could directly affect photosynthesis [34,35]. The conservation of these amino acid residues related to transport of substrates (NPA motifs and ar/R residues) in banana PIPs suggests that these proteins may function on water absorption, plant hydraulics and/or CO_2_ diffusion. Although all MaTIPs and MaNIPs had two identical NPA motifs, some of them showed differences in the ar/R selectivity filter (Table S4, Figures S1, S3 and S4). TIPs are mainly located in vacuolar membranes is involved in the regulation of osmotic potential and water flow across this sub-cellular compartment [33]. Several studies have revealed that TIPs could transport various small solutes, including H_2_O_2_, NH4^+^, and urea, besides water [36,37,38,39]. Additionally, NIPs were also reported to have the ability of transport a wide variety of small solutes, such as water, urea, glycerol, silicon, boric acid, and lactic acid [40,41,42,43,44]. The variation of MaTIPs and MaNIPs in the ar/R selectivity filter sequences may contribute to their multiple transport function.

### 3.2. AQP Genes Are Involved in Fruit Development and Postharvest Ripening of Banana

Because fruit development and ripening processes determine the quality and shelf life of bananas, scientific interest has focused on the study of mechanisms involved in banana development and ripening [45]. Although accumulated evidence has suggested that AQP genes take part in various plant growth and development processes, including seed germination, cell elongation, fruit development, and ripening [19,20,21,28,46], it is unclear whether AQP genes are involved in banana development and ripening. In the present study, we examined expression profiles of MaAQP genes during fruit development and ripening in two cultivated banana varieties (Figure 6, Table S9). The results showed that the majority of MaAQP genes showed transcriptional accumulation, and a large number of MaAQP genes exhibited high expression levels (value > 10) during these stages, indicating the involvement of MaAQP genes in banana fruit development and ripening. In tomato, expression of 32 *AQPs* was examined in different vegetative tissues and developing fruits, among which 23 AQP genes showed transcriptional changes at various developmental stages [28]. In grapes, coincident with the lower xylem hydraulic resistance of the berry, some PIP genes peaked at transcriptional levels during 60 to 80 days after anthesis, and increases in xylem hydraulic resistance beginning 80 days after anthesis corresponded to decreases in expression of *VvPIP1-3* and *VvPIP2-1* [46]. In strawberries, the transcriptional accumulation of *FaPIP1* and *FaPIP2* was higher in the firmer rather than the softer cultivar during fruit ripening, indicating the possible roles of *AQPs* in fruit firmness [47]. Taken together, these investigations indicate that AQP genes extensively participate in the fruit development and ripening process.

In tomato, some PIP and TIP genes, such as *SlPIP1;2*, *;3*, *;5*, and *;7*, *SlPIP2;1*, *;4*, *;6*, *;8*, and *;9*, and *SlTIP1;1* and *SlTIP2;1* in fruit maintained high expression levels from 3 to 14 days after pollination but decreased in the remaining developmental stages [28]. During banana fruit development, we observed that the number of MaAQP genes showing strong transcriptional accumulation (value > 10) at 0 and 20 DAF was significantly greater than that at 80 DAF. Moreover, expression of some MaAQP genes (19/40 in BX; 22/40 in FJ) in all four subfamilies was strong at 0 to 20 DAF but decreased 80 DAF (Figure 6, Table S9). Thus, AQP members in PIPs, TIPs, NIPs, and SIPs may play a crucial role in early fruit development. In plants, apoplastic and symplastic pathways are required for water movement, in which the latter is more efficient at controlling water transport across membranes. AQP family genes play crucial roles in symplastic pathway-mediated water transport [22,48]. Therefore, the transcriptional accumulation of some AQP genes during early fruit development indicates that water movement at inter- and intracellular levels between the cytosol and vacuole are required for fruit development.

Banana is a typical climacteric fruit, undergoing a series of postharvest ripening processes that give rise to numerous physiological and biochemical changes that lead to the quality formation of fruit [49]. Although previous reports have shown that AQP genes are involved in fruit ripening, less information is available for AQP genes in postharvest fruit ripening [28,50]. In this study, we examined changes in the expression of MaAQP genes during postharvest banana ripening and found that most AQP genes showed transcriptional accumulation. Interestingly, the number of MaAQP genes with high expression levels (value > 10) was significantly less at 8 and 14 DPH than that at 80 DAF in BX, whereas this phenomenon was not observed in FJ. Moreover, the expression of some AQP genes (22/40) significantly decreased at 8 and 14 DPH compared to the 80 DAF stage in BX, and only *MaTIP4-2* displayed this expression pattern in FJ. We also noticed that some AQP genes (22/40) showed higher expression levels at 3 DPH compared with 80 DAF in FJ (Figure 6, Table S9). Collectively, these results implied that water movement may be more active in FJ than in BX during postharvest banana ripening process. Notably, we also observed that FJ ripened faster than BX during postharvest ripening. It took 8 and 14 DPH to reach more green than yellow and full yellow degrees of ripening for BX, respectively, whereas it only took 3 and 6 DPH for FJ, respectively. Therefore, AQP-mediated water movement may be involved in acceleration of postharvest banana ripening.

### 3.3. AQP Genes Are Involved in Banana Responses to Abiotic Stress

Because banana plants have shallow roots, a permanent green canopy, and rapid growth rate, they are considered to be extremely sensitive to water stress induced by drought, salt, cold, and other environmental stresses, and hence need an abundant water supply during growth and development [51]. Environmental stresses do great harm to banana growth and development and constrain its production. For the genetic improvement of stress resistance in banana, it is necessary to understand the molecular mechanisms of its abiotic stress responses. Previously, biochemical and genetic evidence has demonstrated that some AQP genes, including *TaAQP7*, *TaAQP8*, *TaNIP*, *MaPIP1;1*, *MusaPIP2;6*, and *NtAQP1*, confer plants resistance to abiotic stress [3,22,23,24,25,52], indicating positive roles for AQP genes during abiotic stress. In this study, we found a large number of MaAQP genes showed transcriptional changes when responding to cold, salt, and osmotic stresses both in BX and FJ, suggesting an extensive response of MaAQP genes to abiotic stress. Moreover, some MaAQP genes, such as *MaPIP2-6*, *MaTIP4-3*, *MaTIP2-1*, *MaTIP2-3*, *MaTIP1-4*, *MaTIP1-5* and *MaTIP4-2*, showed strong induction (*p* < 0.05; log2-based value > 2) after stress treatment and therefore, may represent potential candidates for improving banana resistance to abiotic stress (Figure 7, Tables S10 and S11).

Additionally, we noticed that the number of upregulated MaAQP genes was greater in FJ than in BX after salt and osmotic treatments, and some AQP genes displayed differential expression patterns in both varieties in response to both stresses. *MaPIP2-6*, *MaNIP2-1*, *MaSIP2-1*, *MaTIP3-2*, *MaTIP2-3*, *MaTIP2-4*, and *MaTIP1-4* showed upregulation in FJ, but downregulation or no transcriptional changes in BX after salt and osmotic treatments (Figure 7, Tables S10 and S11). Cultivated bananas originated either from intraspecific hybridizations between wild diploid subspecies of *M. acuminata* (A-genome) or from interspecific crosses between *M. acuminata* and wild diploid *M. balbisiana* (B-genome). The B-genome (*M. balbisiana*) has been associated with improved vigor and tolerance to biotic and abiotic stresses and is thus a target for banana breeding programs [53]. Moreover, most banana varieties containing the B-genome display strong resistance to water stress [54,55]. Before transcriptomic analyses, we evaluated the resistance of the two banana varieties to salt and osmotic stresses and showed that FJ are more tolerant to these stresses compared with BX. Therefore, some genes localized on the B-genome of FJ might contribute to regulation of AQP gene expression, thus leading to their upregulation and improved resistance of FJ to salt and osmotic stresses. This suggests that AQP-mediated water movements at inter- and intracellular levels are essential for improving banana resistance to salt and osmotic stresses.

## 4. Experimental Section

### 4.1. Plant Materials and Treatments

Young banana seedlings of two cultivated varieties, BaXi Jiao (*Musa acuminate* L. AAA group cv. Cavendish; hereafter referred to as BX) and Fen Jiao (*Musa* ABB Pisang Awak; hereafter referred to as FJ), with consistent growth states at the five-leaf stage were obtained from the banana tissue culture center (Danzhou, Institute of Banana and Plantain, Chinese Academy of Tropical Agricultural Sciences). Banana seedlings grew in soil under a growth chamber (28 °C; 200 μmol·m^−2^·s^−1^ light intensity; 16-h light/8-h dark cycle; 70% relative humidity) or in a banana planting base (Wenchang, Institute of Tropical Bioscience and Biotechnology, Chinese Academy of Tropical Agricultural Sciences, Guangzhou, China).

For expression assay in different organs, leaves and roots of banana seedlings at the five-leaf stage and fruits at 80 days after flowering (DAF) were collected to examine the transcriptional accumulation of AQP genes in the two banana varieties. During the fruit development process, bananas at 0, 20, and 80 DAF represent coming out of the bud, cutting flower, and full-grown/harvest stages, respectively. For expression analysis of MaAQP genes during fruit development, fruits at 0, 20, and 80 DAF were collected from BX and FJ. As indicated by Pua *et al.* [56], the degrees of postharvest banana ripening were divided into seven stages, namely full green, trace yellow, more green than yellow, more yellow than green, green tip, full yellow, and yellow flecked with brown spots. It took 8 and 14 days postharvest (DPH) for BX to reach the more green than yellow and full yellow stages, respectively, but only 3 and 6 DPH for FJ, respectively. Thus, for expression analysis in postharvest banana ripening processes, fruits at 8 and 14 DPH in BX and 3 and 6 DPH in FJ were collected to test MaAQP gene transcripts. For expression analysis in response to cold, banana seedlings at the five-leaf stage were placed in a chamber with low temperature (4 °C) for 22 h. For expression analysis in response to salt and osmotic stresses, banana seedlings at the five-leaf stage were irrigated with 300 mM NaCl and 200 mM mannitol for 7 days, respectively. Then, the leaves of banana seedlings were sampled to detect expression patterns of MaAQP genes. Each sample contains two biological replicates.

### 4.2. Identification and Phylogenetic Analyses

The banana genome database was used to obtain DNA and protein sequences [57]. To predict AQP proteins of banana, a Hidden Markov Model built from known AQPs was employed to search the banana database [58]. Additionally, BLAST analyses with all AQPs from *Arabidopsis* and rice as queries were used to further identify possible AQPs in the banana database. RGAP and UniPort databases were adopted to obtain AQP amino acid sequences of rice and *Arabidopsis*, respectively [59,60]. All identified AQPs were further validated by conserved domain searching using CDD and PFAM databases [61,62]. A bootstrap neighbor-joining phylogenetic tree was created based on multiple alignments of the identified AQPs from banana with all the AQPs from *Arabidopsis* and rice using Clustal X 2.0 and MEGA 5.0 with 1000 bootstraps [63,64]. The sequences of banana AQP genes identified in this study could be available in banana genome database [57] (The accession numbers were listed in Table S2).

### 4.3. Protein Properties and Sequence Analyses

The ExPASy proteomics server database was used to predict relative molecular weights (RMW) and isoelectric points (pI) of the identified MaAQP proteins [65]. TMHMM Server ver.2.0 was used to predict the transmembrane domains of MaAQPs [66]. The NPA motif and sequences of ar/R selectivity filter were predicted based on multiple sequence alignment with DNAMAN. MaAQP protein motifs were analyzed with MEME software [67]. The optimum width of motifs ranged from 11 to 50, with the maximum number of motifs being 15; other parameter settings used were default values [5]. The predicted motifs of MaAQP proteins were further annotated with an InterProScan database search [68]. Gene structures of *MaAQPs* were determined with GSDS software based on the genome and coding sequences of *MaAQPs* [69].

### 4.4. Transcriptome Analysis

Expression patterns of MaAQP genes during development, ripening, and abiotic stress were examined with RNA sequencing technique. Different tissues from leaves, roots, and fruits at different developmental and ripening stages, as well as leaves of banana under normal or abiotic stress treatments in BX and FJ were sampled to extract total RNA using a plant RNA extraction kit (Tiangen, Beijing, China). Total RNA (3 μg) from each sample was converted into cDNA using a RevertAid First Strand cDNA Synthesis kit (Fermentas, Waltham, MA, USA). cDNA libraries were constructed based on Illumina protocols and subsequently subjected to sequencing by Illumina GAII sequencing platform following the Illumina RNA sequencing protocol. The average read size of the library was approximately 200 bp and both ends of the cDNAs were sequenced. The raw reads were cleaned by removing adapter sequences, empty reads, and low quality sequences (reads with unknown base pairs “N”). All the downstream analyses were based on the clean data with high quality. The genome sequence of DH-Pahang (*Musa acuminate*, A-genome, 2*n* = 22) was used as a reference to analyze transcriptomic data [57]. Reference genome and gene model annotation files were downloaded from the banana genome website [57] directly. Index of the reference genome was built using Bowtie v1.1.1 [70] and paired end clean reads were aligned to the reference genome using TopHat v2.0.13 [71]. HTSeq v0.6.1 was used to count the reads numbers mapped to each gene [72]. In addition, then FPKM of each gene was calculated based on the length of the gene and reads count mapped to this gene: FPKM = total exon fragments/[mapped reads (millions) × exon length (kb)]. Statistical analysis was performed based on Student’s *t*-test (*n* = 2). Each sample contained two biological replicates.

### 4.5. qRT-PCR Analysis

Changes in the expression of MaAQP genes in different tissues, different stages of fruit development and ripening, and response to abiotic stress were detected by quantitative real-time polymerase chain reaction (qRT-PCR) analysis using SYBR® Premix Ex Taq™ (TaKaRa, Shiga, Japan) chemistry on a Stratagene Mx3000P (Stratagene, CA, USA) instrument. Prior to quantification experiments, a series of primer and template dilutions were conducted to measure the optimal primer and template concentrations. Primers with high specificity and efficiency determined by melting curve analysis and agarose gel electrophoresis were selected to perform quantification assay (Table S12). Furthermore, the specificity for each primer pairs was confirmed after sequence analysis of the resulted products. Amplification efficiencies of primer pairs were between 0.9 and 1.1. *MaRPS2* (HQ853246) and *MaUBQ2* (HQ853254) verified to be constitutive expression and suitable as internal controls were employed as reference genes to normalize the relative expression of target genes [73]. The relative expression levels of the target genes were assessed based on 2^–ΔΔ*C*t^ method [74]. Each sample contains three replicates.

## 5. Conclusions

This systematic analysis identified 47 AQP genes in the banana genome, which was further supported by phylogenetic, gene structural, and conserved protein motif analyses. Expression analysis of MaAQP genes suggested the involvement of MaAQP genes in fruit development, postharvest ripening, and abiotic stress responses. Interestingly, we found some MaAQP genes may function on promoting early fruit development, accelerating postharvest banana ripening processes, and improving plant resistance to salt and osmotic stresses by comparing the expression profiles of MaAQP genes in two banana varieties. These data bring new insight to the control of MaAQP gene expression at the transcriptional level, which provides new clues for further functional characterization of AQP genes and genetic improvement of bananas.

## Supplementary Materials

Supplementary materials can be found at http://www.mdpi.com/1422-0067/16/08/19728/s1.
