# Supplementary material for: Genome-Wide Identification and Expression Analyses of Aquaporin Gene Family during Development and Abiotic Stress in Banana
Source: Int J Mol Sci. 2015 Aug 20;16(8):19728–51. doi: 10.3390/ijms160819728 (PMC4581322; doi:10.3390/ijms160819728)
Supplement: Supplementary file 1 [file ijms-16-19728-s001.zip › ijms-91622-Supplementary Information/ijms-91622-Supplementary-Figures and Tables S1,S2.pdf]

Supplementary Information

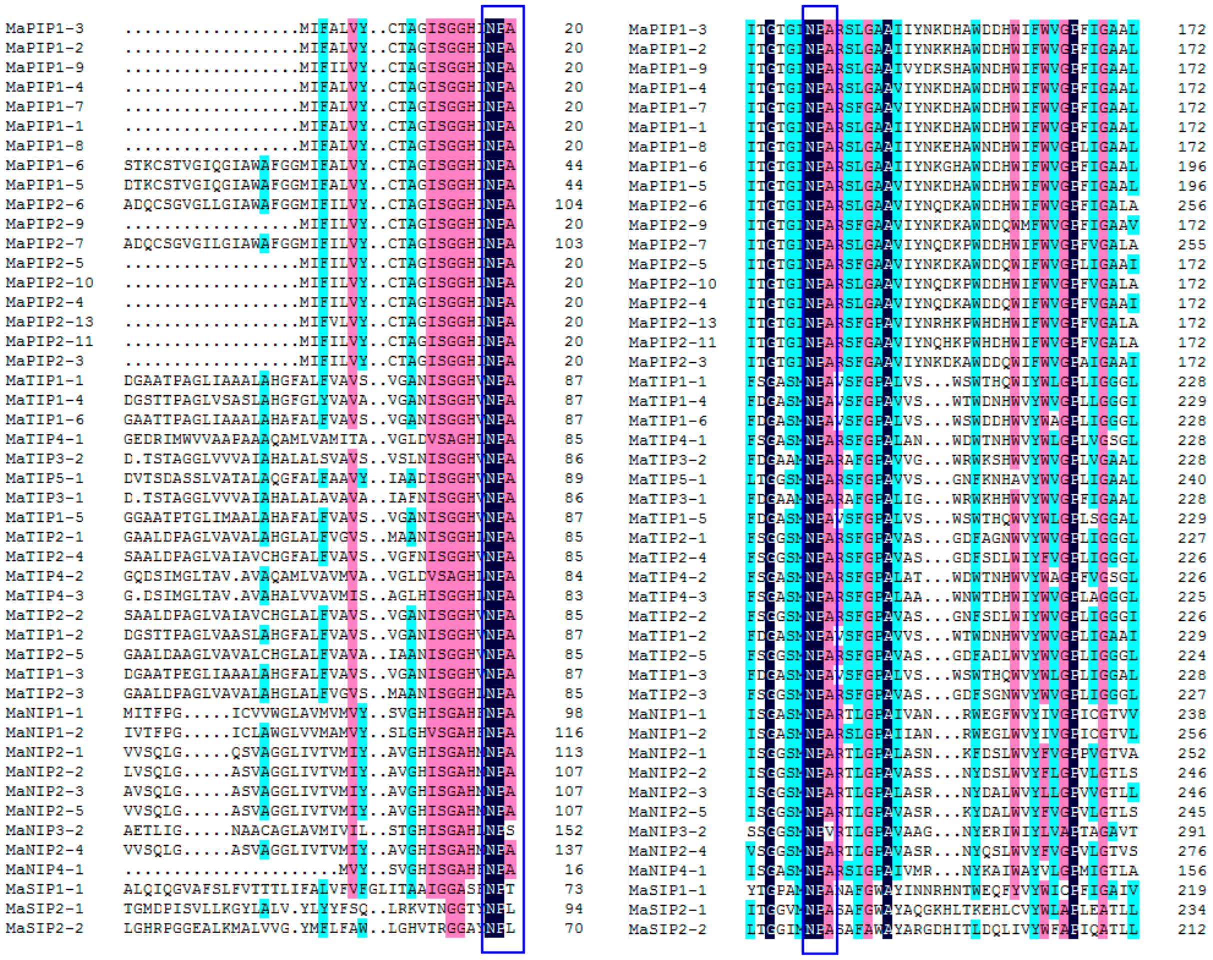

Figure S1. Alignment of amino acid sequences of MaAQPs. The two conserved NPA motifs are shown in blue box. Amino acids with high identities were shown in black background.

```
SlPIP1-1     MAENKEEDVNLGANKFREPQPLGTSAQTDKDYKEPPPAPL   40
MaPIP1-3     ........................................    0
MaPIP1-2     ........................................    0
MaPIP1-9     ........................................    0
MaPIP1-4     ........................................    0
MaPIP1-7     ........................................    0
MaPIP1-1     ........................................    0
MaPIP1-8     ........................................    0
MaPIP1-6     ........................................    0
MaPIP1-5     ........................................    0
MaPIP2-6     ...............MSKEVSVEVEQPPAKDYSDPPPAPL   25
MaPIP2-9     ........................................    0
MaPIP2-7     ...............MSKEVS.EAEQAPAKDYRDPPPAPL   24
MaPIP2-5     ........................................    0
MaPIP2-10    ........................................    0
MaPIP2-4     ........................................    0
MaPIP2-13    ........................................    0
MaPIP2-11    ........................................    0
MaPIP2-3     ........................................    0
Consensus

SlPIP1-1     YEFGELSSWSFYRAGIAEFMATFLFLYITILTVMG..LKR   78
MaPIP1-3     ........................................    0
MaPIP1-2     ........................................    0
MaPIP1-9     ........................................    0
MaPIP1-4     ........................................    0
MaPIP1-7     ........................................    0
MaPIP1-1     ........................................    0
MaPIP1-8     ........................................    0
MaPIP1-6     .................................MG..VVK    5
MaPIP1-5     .................................MG..VVK    5
MaPIP2-6     LDFGEVRLWSFYRALIAEFVATLLFLYVSIATVIGHKEQN   65
MaPIP2-9     ........................................    0
MaPIP2-7     LDFGELRLWSFYRALIAEFVATLLFLYVTIATVIGHKEQN   64
MaPIP2-5     ........................................    0
MaPIP2-10    ........................................    0
MaPIP2-4     ........................................    0
MaPIP2-13    ........................................    0
MaPIP2-11    ........................................    0
MaPIP2-3     ........................................    0

                                 H2
SlPIP1-1     SDSLCSSVGIQGVAWAFGGMIFALVYCTAGISGGHINPAV  118
MaPIP1-3     ...................MIFALVYCTAGISGGHINPAV   21
MaPIP1-2     ...................MIFALVYCTAGISGGHINPAV   21
MaPIP1-9     ...................MIFILVYCTAGISGGHINPAV   21
MaPIP1-4     ...................MIFALVYCTAGISGGHINPAV   21
MaPIP1-7     ...................MIFALVYCTAGISGGHINPAV   21
MaPIP1-1     ...................MIFALVYCTAGISGGHINPAV   21
MaPIP1-8     ...................MIFALVYCTAGISGGHINPAV   21
MaPIP1-6     SSTKCSTVGIQGIAWAFGGMIFALVYCTAGISGGHINPAV   45
MaPIP1-5     SDTKCSTVGIQGIAWAFGGMIFALVYCTAGISGGHINPAV   45
MaPIP2-6     AADQCSGVGLLGIAWAFGGMIFILVYCTAGISGGHINPAV  105
MaPIP2-9     ...................MIFILVYCTAGISGGHINPAV   21
MaPIP2-7     AADQCSGVGILGIAWAFGGMIFILVYCTAGISGGHINPAV  104
MaPIP2-5     ...................MIFILVYCTAGISGGHINPAV   21
MaPIP2-10    ...................MIFILVYCTAGISGGHINPAV   21
MaPIP2-4     ...................MIFILVYCTAGISGGHINPAV   21
MaPIP2-13    ...................MIFVLVYCTAGISGGHINPAV   21
MaPIP2-11    ...................MIFILVYCTAGISGGHINPAV   21
MaPIP2-3     ...................MIFILVYCTAGISGGHINPAV   21
```

Figure S2. *Cont.*

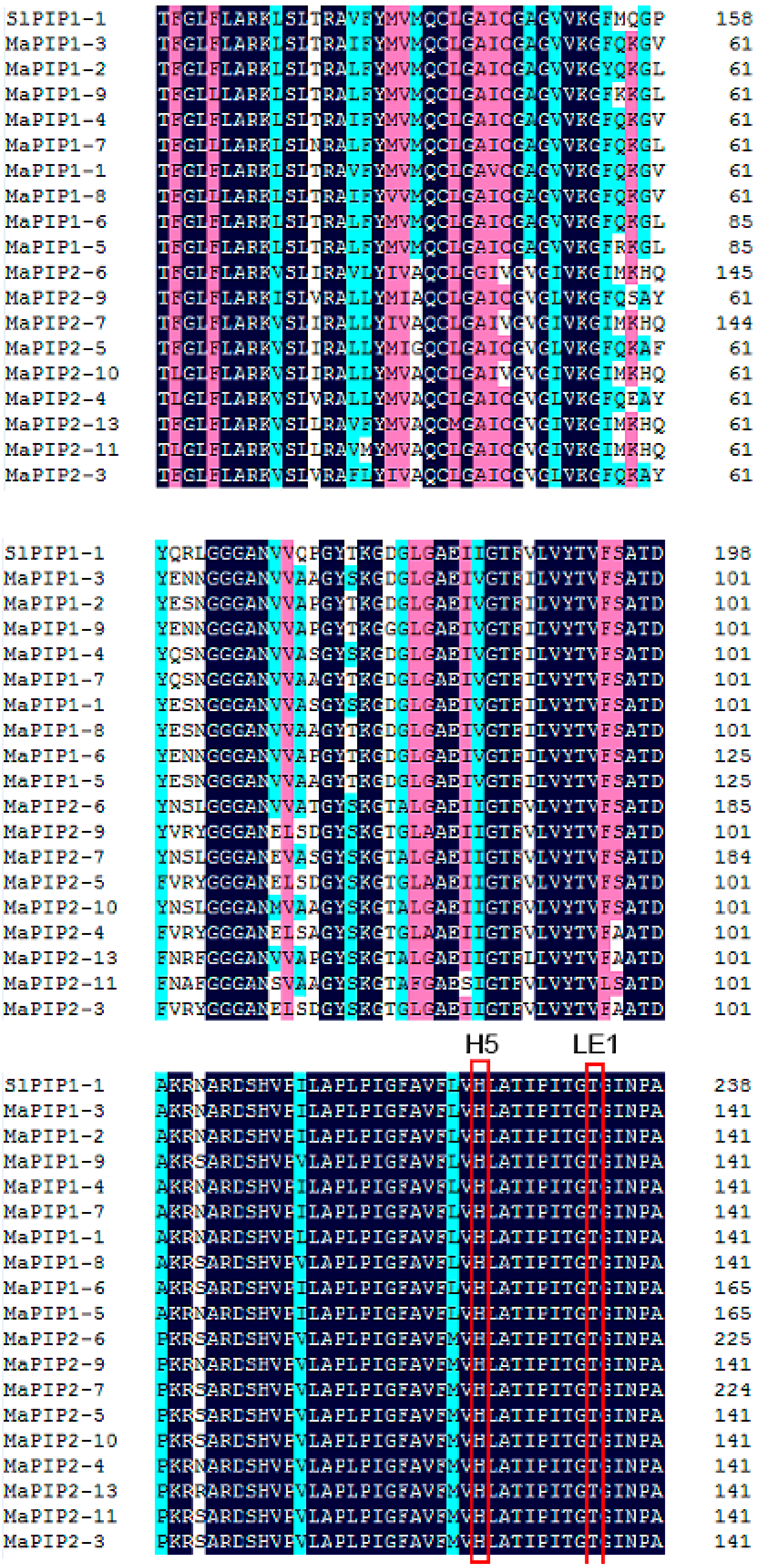

Figure S2. *Cont.*

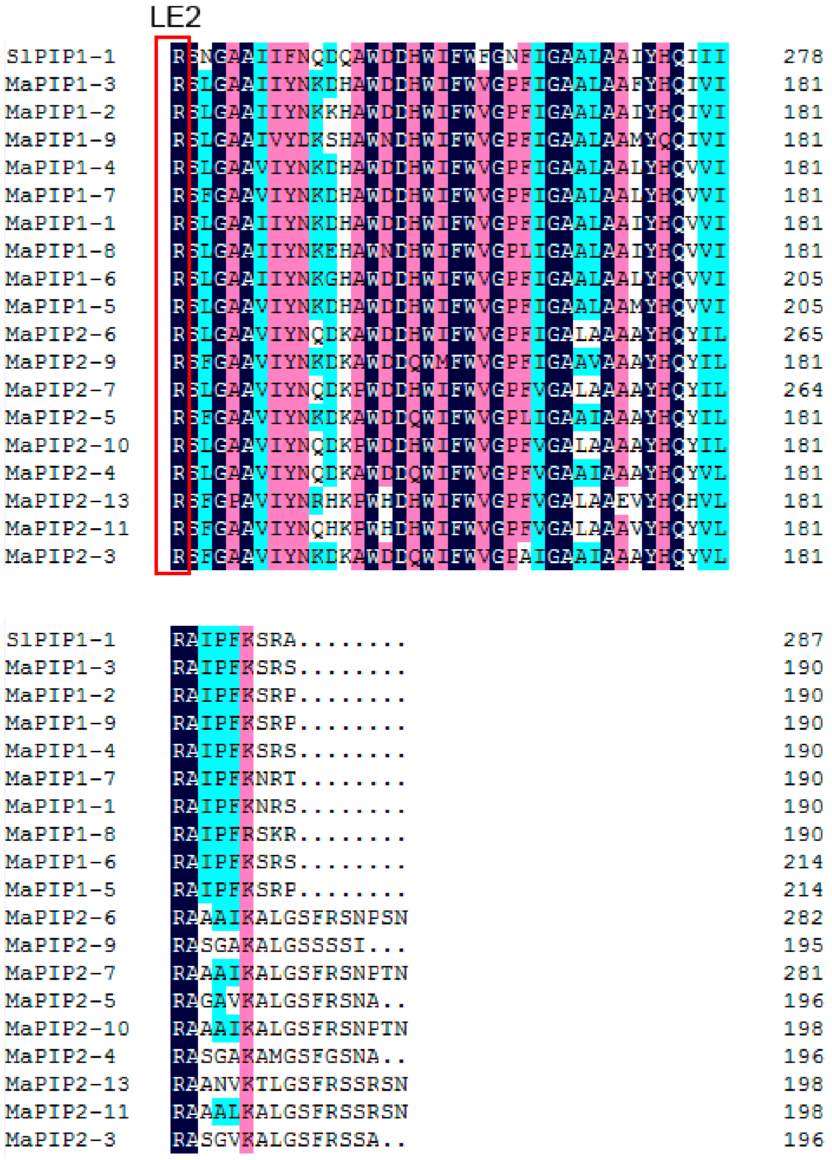

Figure S2. Alignment of amino acid sequences of MaPIPs. Residues comprising the ar/R filter are marked in red box and labeled as H2, H5, LE1 and LE2. Amino acids with high identities were shown in black background.

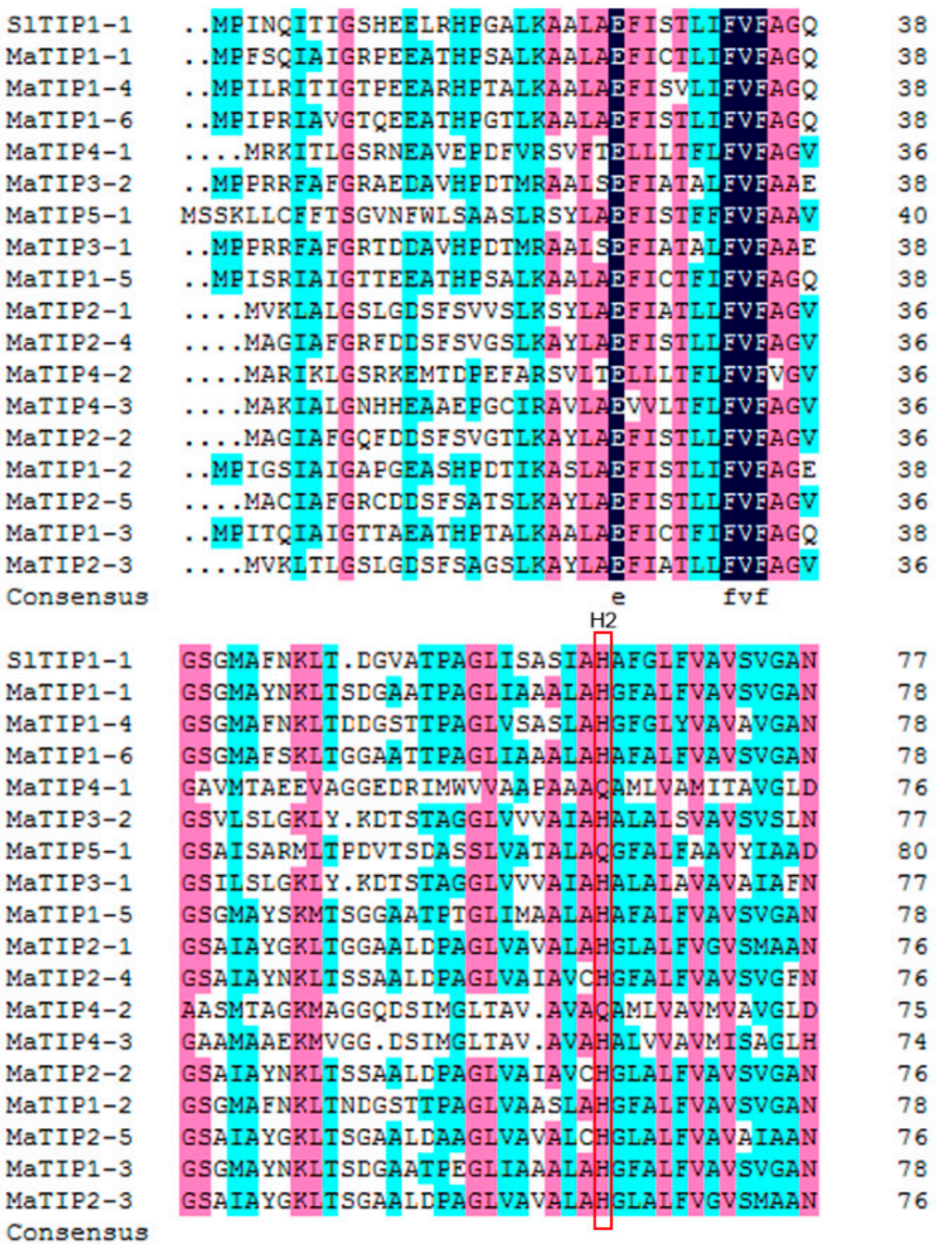

Figure S3. *Cont.*

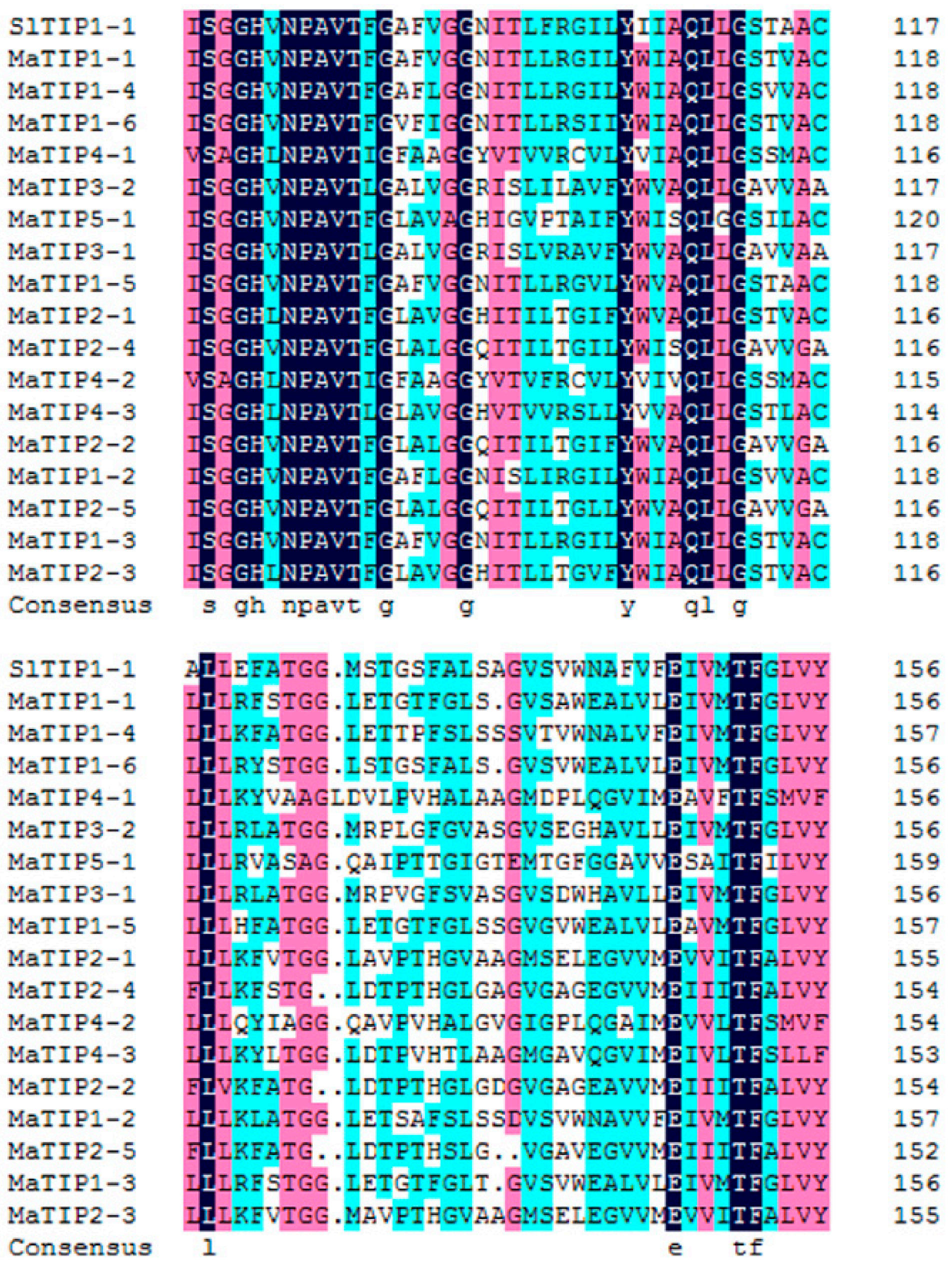

Figure S3. *Cont.*

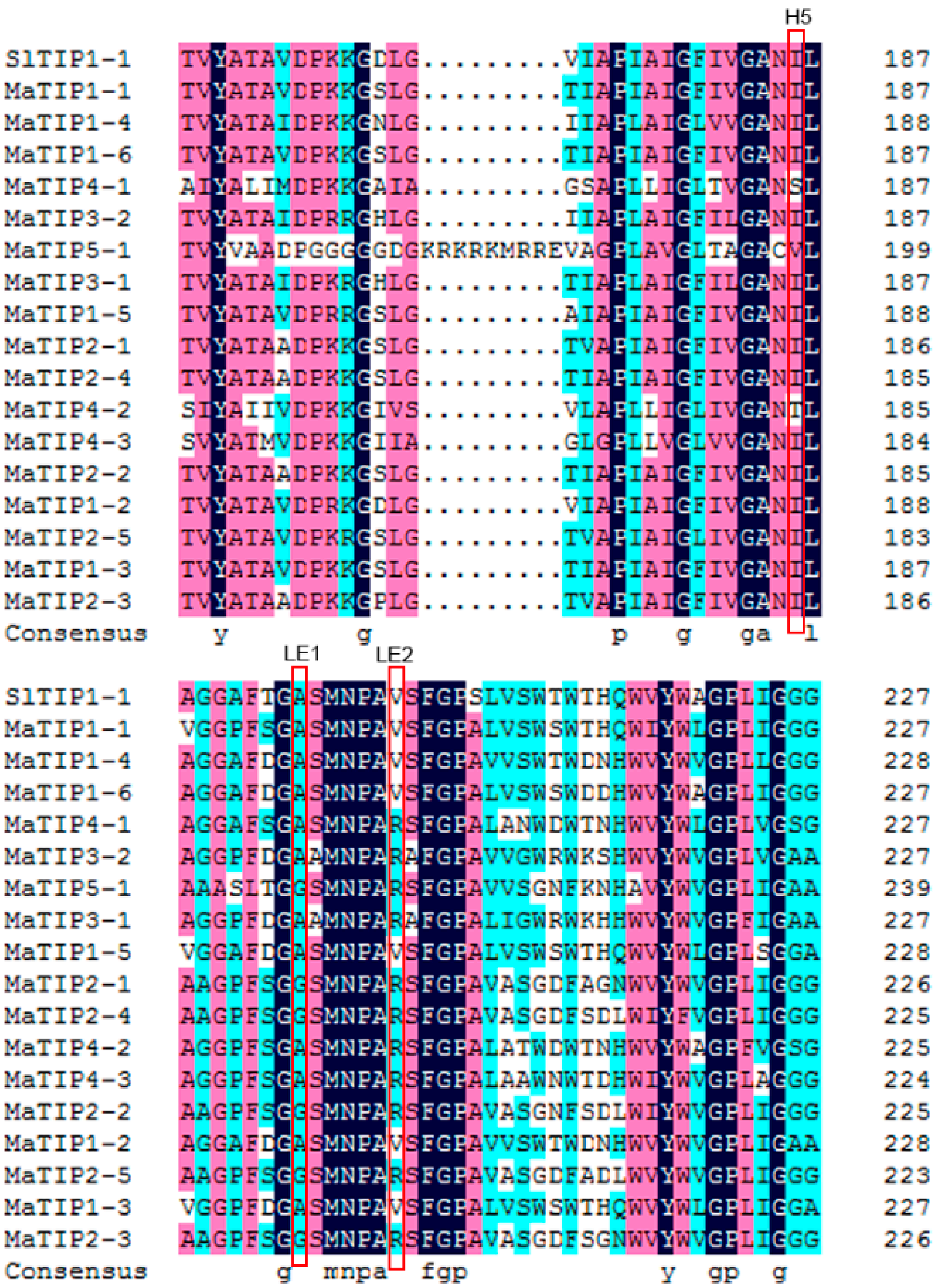

Figure S3. *Cont.*

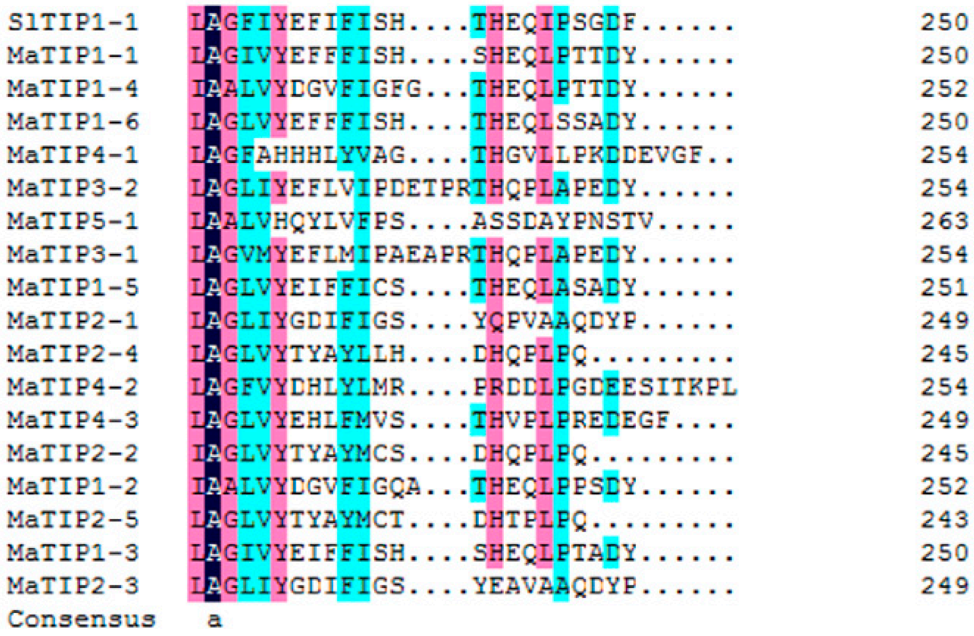

Figure S3. Alignment of amino acid sequences of MaTIPs. Residues comprising the ar/R filter are marked in red box and labeled as H2, H5, LE1 and LE2. Amino acids with high identities were shown in black background.

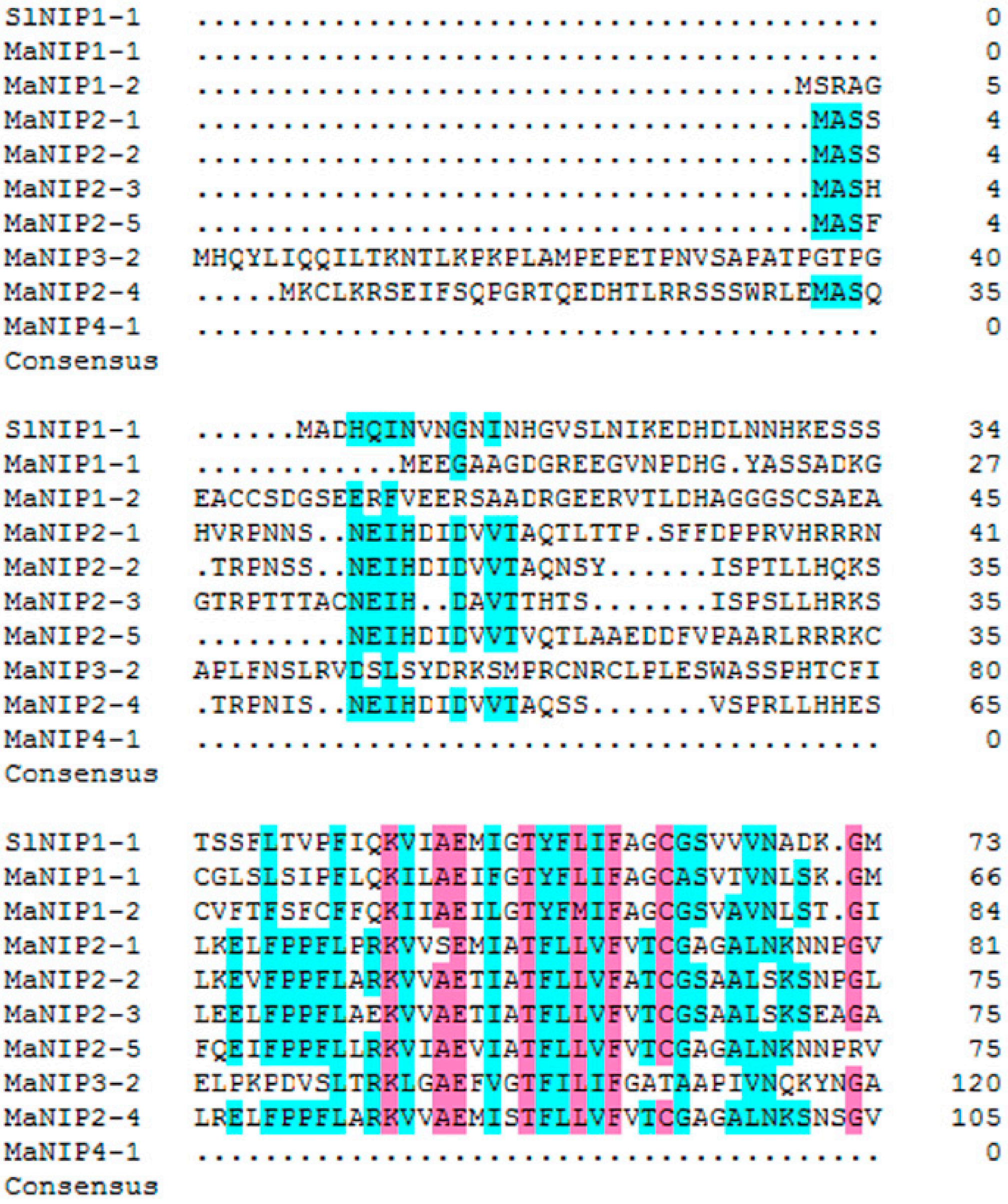

Figure S4. *Cont.*

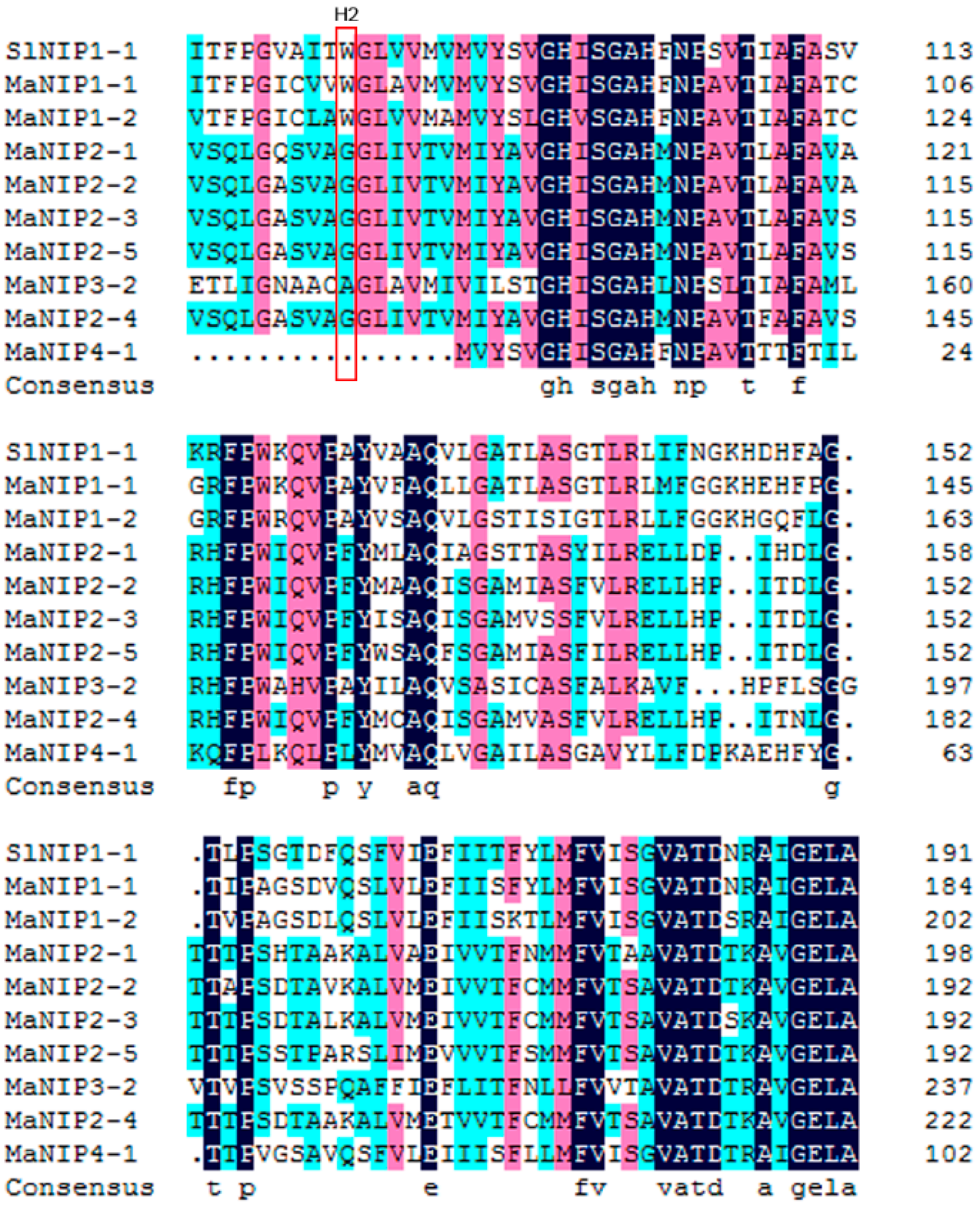

Figure S4. *Cont.*

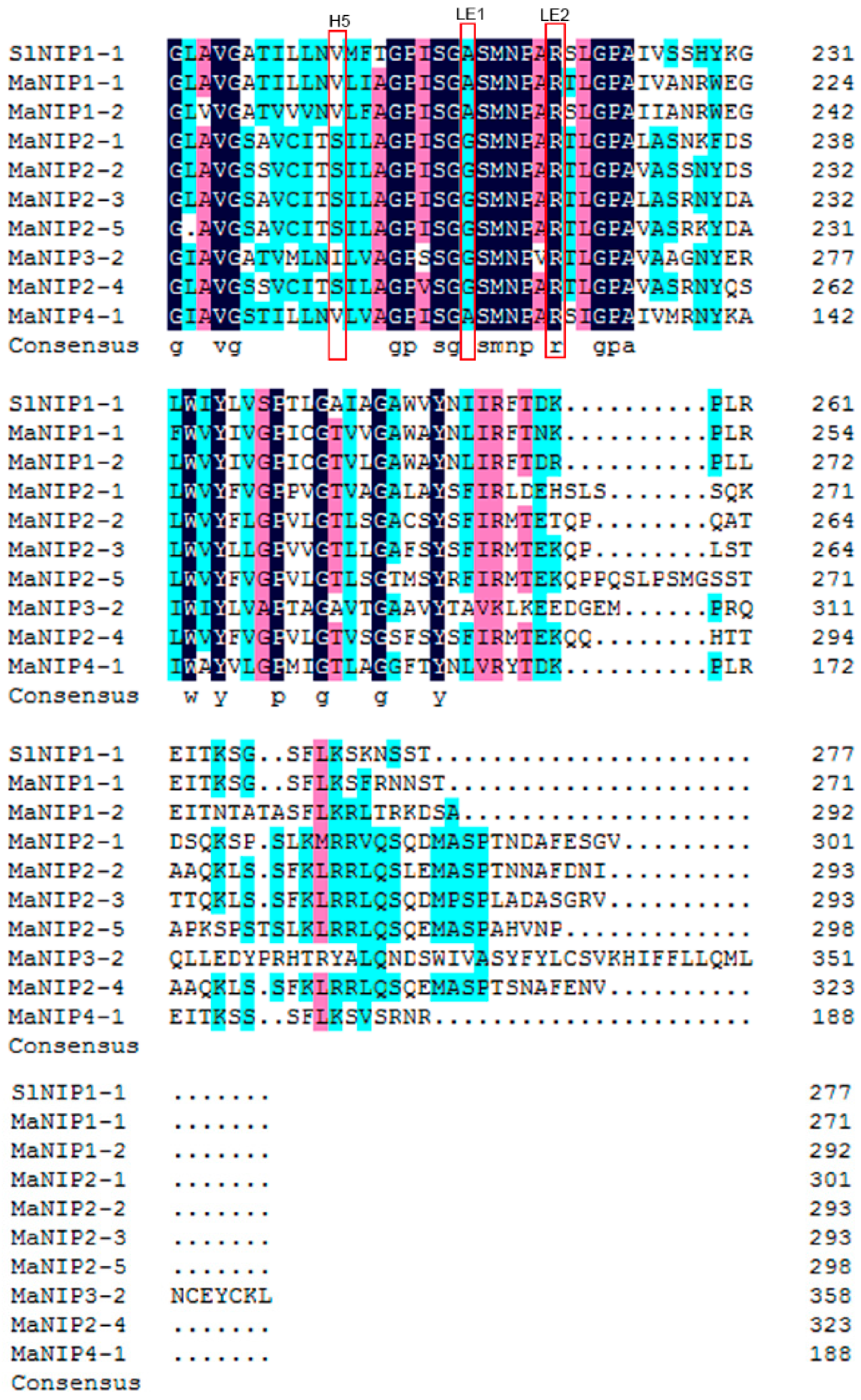

Figure S4. Alignment of amino acid sequences of MaNIPs. Residues comprising the ar/R filter are marked in red box and labeled as H2, H5, LE1 and LE2. Amino acids with high identities were shown in black background.

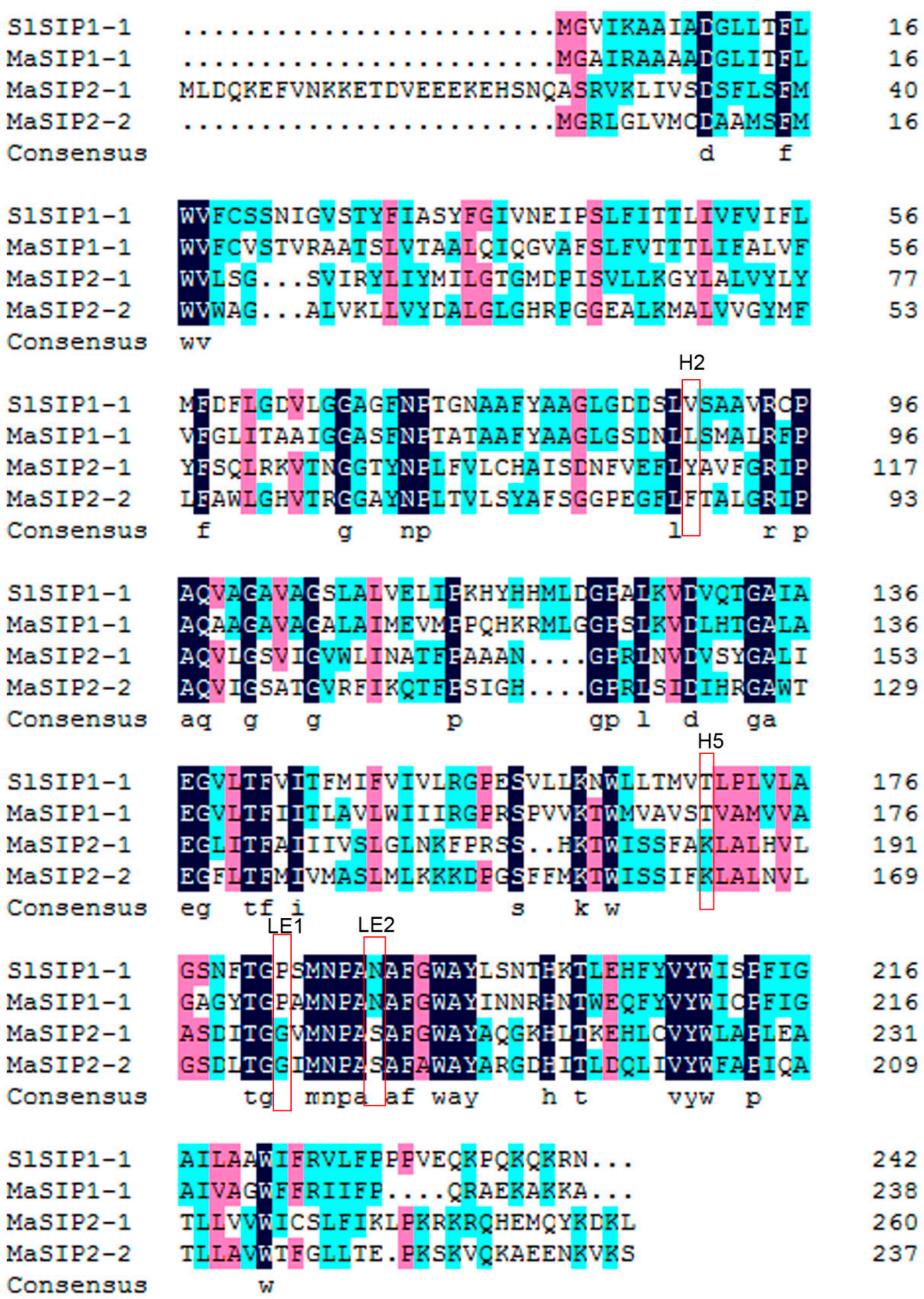

Figure S5. Alignment of amino acid sequences of MaSIPs. Residues comprising the ar/R filter are marked in red box and labeled as H2, H5, LE1 and LE2. Amino acids with high identities were shown in black background.

Table S1. The amino acids of banana AQPs.

>MaPIP1-3

MIFALVYCTAGISGGHINPAVTFGLFLARKLSLTRAIFYMVMQCLGAICGAGVVKGFQKGVYENNGGGANVVAAG
YSKGDGLGAEIVGTFILVYTVFSATDAKRNARDSHVPILAPLPIGFAVFLVHLATIPITGTGINPARSLGAAIIYNKDHA
WDDHWIFWVGPFIGAALAAFYHQIVIRAIPFKSRS

>MaPIP1-2

MIFALVYCTAGISGGHINPAVTFGLFLARKLSLTRALFYMVMQCLGAICGAGVVKGYQKGLYESNGGGANVVAPG
YTKGDGLGAEIVGTFILVYTVFSATDAKRNARDSHVPILAPLPIGFAVFLVHLATIPITGTGINPARSLGAAIIYNKKH
AWDDHWIFWVGPFIGAALAAIYHQIVIRAIPFKSRP

>MaPIP1-9

MIFILVYCTAGISGGHINPAVTFGLLLARKLSLTRALFYMVMQCLGAICGAGVVKGFKKGLYENNGGGANVVAPG
YTKGGGLGAEIVGTFILVYTVFSATDAKRSARDSHVPVLAPLPIGFAVFLVHLATIPITGTGINPARSLGAAIVYDKSH
AWNDHWIFWVGPFIGAALAAMYQQIVIRAIPFKSRP

>MaPIP2-9

MIFILVYCTAGISGGHINPAVTFGLFLARKISLVRALLYMIAQCLGAICGVGLVKGFQSAYYVRYGGGANELSDGYS
KGTGLAAEIIGTFVLVYTVFSATDPKRNARDSHVPVLAPLPIGFAVFMVHLATIPITGTGINPARSFGAAVIYNKDKA
WDDQWMFWVGPFIGAAVAAAYHQYILRASGAKALGSSSSI

>MaPIP1-4

MIFALVYCTAGISGGHINPAVTFGLFLARKLSLTRAIFYMVMQCLGAICGAGVVKGFQKGVYQSNGGGANVVASG
YSKGDGLGAEIVGTFILVYTVFSATDAKRNARDSHVPILAPLPIGFAVFLVHLATIPITGTGINPARSLGAAVIYNKDH
AWDDHWIFWVGPFIGAALAALYHQVVIRAIPFKSRS

>MaTIP1-6

MPIPRIAVGTQEEATHPGTLKAALAEFISTLIFVFAGQGSGMAFSKLTGGAATTPAGLIAAALAHAFALFVAVSVGAN
ISGGHVNPAVTFGVFIGGNITLLRSIIYWIAQLLGSTVACLLLRYSTGGLSTGSFALSGVSVWEALVLEIVMTFGLVY
TVYATAVDPKKGSLGTIAPIAIGFIVGANILAGGAFDGASMNPAVSFGPALVSWSWDDHWVYWAGPLIGGGLAGLV
YEFFFISHTHEQLSSADY

>MaPIP2-6

MSKEVSVEVEQPPAKDYSDPPPAPLLDFGEVRLWSFYRALIAEFVATLLFLYVSIATVIGHKEQNAADQCSGVGLLG
IAWAFGGMIFILVYCTAGISGGHINPAVTFGLFLARKVSLIRAVLYIVAQCLGGIVGVGIVKGIMKHQYNSLGGGANV
VATGYSKGTALGAEIIGTFVLVYTVFSATDPKRSARDSHVPVLAPLPIGFAVFMVHLATIPITGTGINPARSLGAAVIY
NQDKAWDDHWIFWVGPFIGALAAAAYHQYILRAAAIKALGSFRSNPSN

>MaNIP3-2

MHQYLIQQILTKNTLKPKPLAMPEPETPNVSAPATPGTPGAPLFNSLRVDSLSYDRKSMPRCNRCLPLESWASSPHT
CFIELPKPDVSLTRKLGAEFVGTFILIFGATAAPIVNQKYNGAETLIGNAACAGLAVMIVILSTGHISGAHLNPSLTIAF
AMLRHFPWAHVPAYILAQVSASICASFALKAVFHPFLSGGVTVPSVSSPQAFFIEFLITFNLLFVVTAVATDTRAVGEL
AGIAVGATVMLNILVAGPSSGGSMNPVRTLGPAVAAGNYERIWIYLVAPTAGAVTGAAVYTAVKLKEEDGEMPRQQ
LLEDYPRHTRYALQNDSWIVASYFYLCSVKHIFFLLQMLNCEYCKLR

>MaTIP4-1

MRKITLGSRNEAVEPDFVRSVFTELLLTFLFVFAGVGAVMTAEEVAGGEDRIMWVVAAPAAAQAMLVAMITAVGL
DVSAGHLNPAVTIGFAAGGYVTVVRCVLYVIAQLLGSSMACLLLKYVAAGLDVLPVHALAAGMDPLQGVIMEAV
FTFSMVFAIYALIMDPKKGAIAGSAPLLIGLTVGANSLAGGAFSGASMNPARSFGPALANWDWTNHWVYWLGPLV
GSGLAGFAHHHLYVAGTHGVLLPKDDEVGF

>MaPIP1-7
MIFALVYCTAGISGGHINPAVTFGLLLARKLSLNRALFYMVMQCLGAICGAGVVKGFQKGLYQSNGGGANVVAAG
YTKGDGLGAEIVGTFILVYTVFSATDAKRNARDSHVPILAPLPIGFAVFLVHLATIPITGTGINPARSFGAAVIYNKDH
AWDDHWIFWVGPFIGAALAALYHQVVIRAIPFKNRT

>MaTIP3-2
MPPRRFAFGRAEDAVHPDTMRAALSEFIATALFVFAAEGSVLSLGKLYKDTSTAGGLVVVAIAHALALSVAVSVSLN
ISGGHVNPAVTLGALVGGRISLILAVFYWVAQLLGAVVAALLLRLATGGMRPLGFGVASGVSEGHAVLLEIVMTFGL
VYTVYATAIDPRRGHLGIIAPLAIGFILGANILAGGPFDGAAMNPARAFGPAVVGWRWKSHWVYWVGPLVGAALA
GLIYEFLVIPDETPRTHQPLAPEDY

>MaPIP2-7
MSKEVSEAEQAPAKDYRDPPPAPLLDFGELRLWSFYRALIAEFVATLLFLYVTIATVIGHKEQNAADQCSGVGILGIA
WAFGGMIFILVYCTAGISGGHINPAVTFGLFLARKVSLIRALLYIVAQCLGAIVGVGIVKGIMKHQYNSLGGGANEV
ASGYSKGTALGAEIIGTFVLVYTVFSATDPKRSARDSHVPVLAPLPIGFAVFMVHLATIPITGTGINPARSLGAAVIYN
QDKPWDDHWIFWVGPFVGALAAAAYHQYILRAAAIKALGSFRSNPTN

>MaPIP2-5
MIFILVYCTAGISGGHINPAVTFGLFLARKVSLIRALLYMIGQCLGAICGVGLVKGFQKAFFVRYGGGANELSDGYS
KGTGLAAEIIGTFVLVYTVFSATDPKRSARDSHVPVLAPLPIGFAVFMVHLATIPITGTGINPARSFGAAVIYNKDKA
WDDQWIFWVGPLIGAAIAAAYHQYILRAGAVKALGSFRSNA

>MaTIP5-1
MSSKLLCFFTSGVNFWLSAASLRSYLAEFISTFFFVFAAVGSAISARMLTPDVTSDASSLVATALAQGFALFAAVYIA
ADISGGHVNPAVTFGLAVAGHIGVPTAIFYWISQLGGSILACLLLRVASAGQAIPTTGIGTEMTGFGGAVVESAITFIL
VYTVYVAADPGGGGGDGKRKRKMRREVAGPLAVGLTAGACVLAAASLTGGSMNPARSFGPAVVSGNFKNHAVY
WVGPLIGAALAALVHQYLVFPSASSDAYPNSTV

>MaTIP3-1
MPPRRFAFGRTDDAVHPDTMRAALSEFIATALFVFAAEGSILSLGKLYKDTSTAGGLVVVAIAHALALAVAVAIAFNI
SGGHVNPAVTLGALVGGRISLVRAVFYWVAQLLGAVVAALLLRLATGGMRPVGFSVASGVSDWHAVLLEIVMTFG
LVYTVYATAIDPKRGHLGTIAPLAIGFILGANILAGGPFDGAAMNPARAFGPALIGWRWKHHWVYWVGPFIGAAL
AGVMYEFLMIPAEAPRTHQPLAPEDY

>MaPIP2-10

MIFILVYCTAGISGGHINPAVTLGLFLARKVSLIRALLYMVAQCLGAIVGVGIVKGIMKHQYNSLGGGANMVAAGY
SKGTALGAEIIGTFVLVYTVFSATDPKRSARDSHVPVLAPLPIGFAVFMVHLATIPITGTGINPARSLGAAVIYNQDKP
WDDHWIFWVGPFVGALAAAAYHQYILRAAAIKALGSFRSNPTN

>MaPIP1-1
MIFALVYCTAGISGGHINPAVTFGLFLARKLSLTRAVFYMVMQCLGAVCGAGVVKGFQKGVYESNGGGANVVASG
YSKGDGLGAEIVGTFILVYTVFSATDAKRNARDSHVPLLAPLPIGFAVFLVHLATIPITGTGINPARSLGAAIIYNKDH
AWDDHWIFWVGPFIGAALAAIYHQVVIRAIPFKNRS

>MaTIP1-5
MPISRIAIGTTEEATHPSALKAALAEFICTFIFVFAGQGSGMAYSKMTSGGAATPTGLIMAALAHAFALFVAVSVGA
NISGGHVNPAVTFGAFVGGNITLLRGVLYWVAQLLGSTAACLLLHFATGGLETGTFGLSSGVGVWEALVLEAVMTF
GLVYTVYATAVDPRRGSLGAIAPIAIGFIVGANILVGGAFDGASMNPAVSFGPALVSWSWTHQWVYWLGPLSGGAL
AGLVYEIFFICSTHEQLASADY

>MaNIP2-3
MASHGTRPTTTACNEIHDAVTTHTSISPSLLHRKSLEELFPPFLAEKVVAETIATFLLVFVTCGSAALSKSEAGAVSQL
GASVAGGLIVTVMIYAVGHISGAHMNPAVTLAFAVSRHFPWIQVPFYISAQISGAMVSSFVLRELLHPITDLGTTTPS
DTALKALVMEIVVTFCMMFVTSAVATDSKAVGELAGLAVGSAVCITSILAGPISGGSMNPARTLGPALASRNYDAL
WVYLLGPVVGTLLGAFSYSFIRMTEKQPLSTTTQKLSSFKLRRLQSQDMPSPLADASGRV

>MaSIP1-1
MGAIRAAAADGLITFLWVFCVSTVRAATSLVTAALQIQGVAFSLFVTTTLIFALVFVFGLITAAIGGASFNPTATAAFY
AAGLGSDNLLSMALRFPAQAAGAVAGALAIMEVMPPQHKRMLGGPSLKVDLHTGALAEGVLTFIITLAVLWIIIRG
PRSPVVKTWMVAVSTVAMVVAGAGYTGPAMNPANAFGWAYINNRHNTWEQFYVYWICPFIGAIVAGWFFRIIFPQ
RAEKAKKA

>MaSIP2-1
MLDQKEFVNKKETDVEEEKEHSNQASRVKLIVSDSFLSFMWVLSGSVIRYLIYMILGTGMDPISVLLKGYLALVYL
YYFSQLRKVTNGGTYNPLFVLCHAISDNFVEFLYAVFGRIPAQVLGSVIGVWLINATFPAAANGPRLNVDVSYGALI
EGLITFAIIIVSLGLNKFPRSSHKTWISSFAKLALHVLASDITGGVMNPASAFGWAYAQGKHLTKEHLCVYWLAPLE
ATLLVVWICSLFIKLPKRKRQHEMQYKDKLV

>MaSIP2-2
MGRLGLVMCDAAMSFMWVWAGALVKLLVYDALGLGHRPGGEALKMALVVGYMFLFAWLGHVTRGGAYNPLT
VLSYAFSGGPEGFLFTALGRIPAQVIGSATGVRFIKQTFPSIGHGPRLSIDIHRGAWTEGFLTFMIVMASLMLKKKDP
GSFFMKTWISSIFKLALNVLGSDLTGGIMNPASAFAWAYARGDHITLDQLIVYWFAPIQATLLAVWTFGLLTEPKSK
VQKAEENKVKSE

>MaNIP2-4
MKCLKRSEIFSQPGRTQEDHTLRRSSSWRLEMASQTRPNISNEIHDIDVVTAQSSVSPRLLHHESLRELFPPFLARKV
VAEMISTFLLVFVTCGAGALNKSNSGVVSQLGASVAGGLIVTVMIYAVGHISGAHMNPAVTFAFAVSRHFPWIQVPF

YMCAQISGAMVASFVLRELLHPITNLGTTTPSDTAAKALVMETVVTFCMMFVTSAVATDTKAVGELAGLAVGSSV
CITSILAGPVSGGSMNPARTLGPAVASRNYQSLWVYFVGPVLGTVSGSFSYSFIRMTEKQQHTTAAQKLSSFKLRRL
QSQEMASPTSNAFENV

>MaTIP2-4
MAGIAFGRFDDSFSVGSLKAYLAEFISTLLFVFAGVGSAIAYNKLTSSAALDPAGLVAIAVCHGFALFVAVSVGFNIS
GGHVNPAVTFGLALGGQITILTGILYWISQLLGAVVGAFLLKFSTGLDTPTHGLGAGVGAGEGVVMEIIITFALVYT
VYATAADPKKGSLGTIAPIAIGFIVGANILAAGPFSGGSMNPARSFGPAVASGDFSDLWIYFVGPLIGGGLAGLVYTY
AYLLHDHQPLPQ

>MaNIP1-1
MEEGAAGDGREEGVNPDHGYASSADKGCGLSLSIPFLQKILAEIFGTYFLIFAGCASVTVNLSKGMITFPGICVVWG
LAVMVMVYSVGHISGAHFNPAVTIAFATCGRFPWKQVPAYVFAQLLGATLASGTLRLMFGGKHEHFPGTIPAGSDV
QSLVLEFIISFYLMFVISGVATDNRAIGELAGLAVGATILLNVLIAGPISGASMNPARTLGPAIVANRWEGFWVYIVGP
ICGTVVGAWAYNLIRFTNKPLREITKSGSFLKSFRNNST

>MaTIP1-4
MPILRITIGTPEEARHPTALKAALAEFISVLIFVFAGQGSGMAFNKLTDDGSTTPAGLVSASLAHGFGLYVAVAVGAN
ISGGHVNPAVTFGAFLGGNITLLRGILYWIAQLLGSVVACLLLKFATGGLETTPFSLSSSVTVWNALVFEIVMTFGLV
YTVYATAIDPKKGNLGIIAPLAIGLVVGANILAGGAFDGASMNPAVSFGPAVVSWTWDNHWVYWVGPLLGGGIAA
LVYDGVFIGFGTHEQLPTTDY

>MaPIP1-8
MIFALVYCTAGISGGHINPAVTFGLLLARKLSLTRAIFYVVMQCLGAICGAGVVKGFQKGVYESNGGGANVVAAG
YTKGDGLGAEIVGTFILVYTVFSATDAKRSARDSHVPVLAPLPIGFAVFLVHLATIPITGTGINPARSLGAAIIYNKEH
AWNDHWIFWVGPLIGAALAAIYHQVVIRAIPFRSKR

>MaTIP1-1
MPFSQIAIGRPEEATHPSALKAALAEFICTLIFVFAGQGSGMAYNKLTSDGAATPAGLIAAALAHGFALFVAVSVGA
NISGGHVNPAVTFGAFVGGNITLLRGILYWIAQLLGSTVACLLLRFSTGGLETGTFGLSGVSAWEALVLEIVMTFGL
VYTVYATAVDPKKGSLGTIAPIAIGFIVGANILVGGPFSGASMNPAVSFGPALVSWSWTHQWIYWLGPLIGGGLAGI
VYEFFFISHSHEQLPTTDY

>MaPIP2-4
MIFILVYCTAGISGGHINPAVTLGLFLARKVSLVRALLYMVAQCLGAICGVGLVKGFQEAYFVRYGGGANELSAGY
SKGTGLAAEIIGTFVLVYTVFAATDPKRNARDSHVPVLAPLPIGFAVFMVHLATIPITGTGINPARSLGAAVIYNQDK
AWDDQWIFWVGPFVGAAIAAAYHQYVLRASGAKAMGSFGSNA

>MaPIP2-13
MIFVLVYCTAGISGGHINPAVTFGLFLARKVSLLRAVFYMVAQCMGAICGVGIVKGIMKHQFNRFGGGANVVAPG
YSKGTALGAEIIGTFLLVYTVFAATDPKRRARDSHVPVLAPLPIGFAVFMVHLATIPITGTGINPARSFGPAVIYNRHK
PWHDHWIFWVGPFVGALAAEVYHQHVLRAANVKTLGSFRSSRSNC

>MaPIP2-11
MIFILVYCTAGISGGHINPAVTLGLFLARKVSLLRAVMYMVAQCLGAICGVGIVKGIMKHQFNAFGGGANSVAAGY
SKGTAFGAESIGTFVLVYTVLSATDPKRSARDSHVPVLAPLPIGFAVFMVHLATIPITGTGINPARSFGAAVIYNQHKP
WHDHWIFWVGPFVGALAAAVYHQYVLRAAALKALGSFRSSRSN

>MaNIP4-1
MVYSVGHISGAHFNPAVTTTFTILKQFPLKQLPLYMVAQLVGAILASGAVYLLFDPKAEHFYGTTPVGSAVQSFVLE
IIISFLLMFVISGVATDTRAIGELAGIAVGSTILLNVLVAGPISGASMNPARSIGPAIVMRNYKAIWAYVLGPMIGTLAG
GFTYNLVRYTDKPLREITKSSSFLKSVSRNR

>MaTIP4-2
MARIKLGSRKEMTDPEFARSVLTELLLTFLFVFVGVAASMTAGKMAGGQDSIMGLTAVAVAQAMLVAVMVAVGLD
VSAGHLNPAVTIGFAAGGYVTVFRCVLYVIVQLLGSSMACLLLQYIAGGQAVPVHALGVGIGPLQGAIMEVVLTFS
MVFSIYAIIVDPKKGIVSVLAPLLIGLIVGANTLAGGPFSGASMNPARSFGPALATWDWTNHWVYWAGPFVGSGLA
GFVYDHLYLMRPRDDLPGDEESITKPLC

>MaTIP2-2
MAGIAFGQFDDSFSVGTLKAYLAEFISTLLFVFAGVGSAIAYNKLTSSAALDPAGLVAIAVCHGLALFVAVSVGANIS
GGHVNPAVTFGLALGGQITILTGIFYWVAQLLGAVVGAFLVKFATGLDTPTHGLGDGVGAGEAVVMEIIITFALVYT
VYATAADPKKGSLGTIAPIAIGFIVGANILAAGPFSGGSMNPARSFGPAVASGNFSDLWIYWVGPLIGGGIAGLVYTY
AYMCSDHQPLPQ

>MaNIP2-1
MASSHVRPNNSNEIHDIDVVTAQTLTTPSFFDPPRVHRRRNLKELFPPFLPRKVVSEMIATFLLVFVTCGAGALNKN
NPGVVSQLGQSVAGGLIVTVMIYAVGHISGAHMNPAVTLAFAVARHFPWIQVPFYMLAQIAGSTTASYILRELLDPI
HDLGTTTPSHTAAKALVAEIVVTFNMMFVTAAVATDTKAVGELAGLAVGSAVCITSILAGPISGGSMNPARTLGPAL
ASNKFDSLWVYFVGPPVGTVAGALAYSFIRLDEHSLSSQKDSQKSPSLKMRRVQSQDMASPTNDAFESGV

>MaPIP1-6
MGVVKSSTKCSTVGIQGIAWAFGGMIFALVYCTAGISGGHINPAVTFGLFLARKLSLTRALFYMVMQCLGAICGAG
VVKGFQKGLYENNGGGANVVAPGYTKGDGLGAEIVGTFILVYTVFSATDAKRSARDSHVPILAPLPIGFAVFLVHL
ATIPITGTGINPARSLGAAIIYNKGHAWDDHWIFWVGPFIGAALAALYHQVVIRAIPFKSRS

>MaTIP1-2
MPIGSIAIGAPGEASHPDTIKASLAEFISTLIFVFAGEGSGMAFNKLTNDGSTTPAGLVAASLAHGFALFVAVSVGANI
SGGHVNPAVTFGAFLGGNISLIRGILYWIAQLLGSVVACLLLKLATGGLETSAFSLSSDVSVWNAVVFEIVMTFGLV
YTVYATAVDPRKGDLGVIAPIAIGFIVGANILAGGAFDGASMNPAVSFGPAVVSWTWDNHWVYWVGPLIGAAIAAL
VYDGVFIGQATHEQLPPSDY

>MaTIP2-5

MACIAFGRCDDSFSATSLKAYLAEFISTLLFVFAGVGSAIAYGKLTSGAALDAAGLVAVALCHGLALFVAVAIAANIS
GGHVNPAVTFGLALGGQITILTGLLYWVAQLLGAVVGAFLLKFATGLDTPTHSLGVGAVEGVVMEIIITFALVYTVY
ATAVDPKRGSLGTVAPIAIGLIVGANILAAGPFSGGSMNPARSFGPAVASGDFADLWVYWVGPLIGGGLAGLVYTYA
YMCTDHTPLPQ

>MaNIP2-2
MASSTRPNSSNEIHDIDVVTAQNSYISPTLLHQKSLKEVFPPFLARKVVAETIATFLLVFATCGSAALSKSNPGLVSQL
GASVAGGLIVTVMIYAVGHISGAHMNPAVTLAFAVARHFPWIQVPFYMAAQISGAMIASFVLRELLHPITDLGTTAP
SDTAVKALVMEIVVTFCMMFVTSAVATDTKAVGELAGLAVGSSVCITSILAGPISGGSMNPARTLGPAVASSNYDSL
WVYFLGPVLGTLSGACSYSFIRMTETQPQATAAQKLSSFKLRRLQSLEMASPTNNAFDNI

>MaTIP4-3
MAKIALGNHHEAAEPGCIRAVLAEVVLTFLFVFAGVGAAMAAEKMVGGDSIMGLTAVAVAHALVVAVMISAGLHI
SGGHLNPAVTLGLAVGGHVTVVRSLLYVVAQLLGSTLACLLLKYLTGGLDTPVHTLAAGMGAVQGVIMEIVLTFSL
LFSVYATMVDPKKGIIAGLGPLLVGLVVGANILAGGPFSGASMNPARSFGPALAAWNWTDHWIYWVGPLAGGGL
AGLVYEHLFMVSTHVPLPREDEGF

>MaPIP2-3
MIFILVYCTAGISGGHINPAVTFGLFLARKVSLVRAFLYIVAQCLGAICGVGLVKGFQKAYFVRYGGGANELSDGYS
KGTGLGAEIIGTFVLVYTVFAATDPKRSARDSHVPVLAPLPIGFAVFMVHLATIPITGTGINPARSFGAAVIYNKDKA
WDDQWIFWVGPAIGAAIAAAYHQYVLRASGVKALGSFRSSA

>MaTIP1-3
MPITQIAIGTTAEATHPTALKAALAEFICTFIFVFAGQGSGMAYNKLTSDGAATPEGLIAAALAHGFALFVAVSVGAN
ISGGHVNPAVTFGAFVGGNITLLRGILYWIAQLLGSTVACLLLRFSTGGLETGTFGLTGVSVWEALVLEIVMTFGLV
YTVYATAVDPKKGSLGTIAPIAIGFIVGANILVGGPFDGASMNPAVSFGPALVSWSWTHQWVYWLGPLIGGALAGIV
YEIFFISHSHEQLPTADY

>MaTIP2-3
MVKLTLGSLGDSFSAGSLKAYLAEFIATLLFVFAGVGSAIAYGKLTSGAALDPAGLVAVALAHGLALFVGVSMAANI
SGGHLNPAVTFGLAVGGHITLLTGVFYWIAQLLGSTVACLLLKFVTGGMAVPTHGVAAGMSELEGVVMEVVITFAL
VYTVYATAADPKKGPLGTVAPIAIGFIVGANILAAGPFSGGSMNPARSFGPAVASGDFSGNWVYWVGPLIGGGLAG
LIYGDIFIGSYEAVAAQDYP

>MaPIP1-5
MGVVKSDTKCSTVGIQGIAWAFGGMIFALVYCTAGISGGHINPAVTFGLFLARKLSLTRALFYMVMQCLGAICGAG
VVKGFRKGLYESNGGGANVVAAGYTKGDGLGAEIVGTFILVYTVFSATDAKRNARDSHVPILAPLPIGFAVFLVHL
ATIPITGTGINPARSLGAAVIYNKDHAWDDHWIFWVGPFIGAALAAMYHQVVIRAIPFKSRP

>MaTIP2-1
MVKLALGSLGDSFSVVSLKSYLAEFIATLLFVFAGVGSAIAYGKLTGGAALDPAGLVAVALAHGLALFVGVSMAAN
ISGGHLNPAVTFGLAVGGHITILTGIFYWVAQLLGSTVACLLLKFVTGGLAVPTHGVAAGMSELEGVVMEVVITFAL

VYTVYATAADPKKGSLGTVAPIAIGFIVGANILAAGPFSGGSMNPARSFGPAVASGDFAGNWVYWVGPLIGGGLAG
LIYGDIFIGSYQPVAAQDYP

>MaNIP1-2
MSRAGEACCSDGSEERFVEERSAADRGEERVTLDHAGGGSCSAEACVFTFSFCFFQKIIAEILGTYFMIFAGCGSVA
VNLSTGIVTFPGICLAWGLVVMAMVYSLGHVSGAHFNPAVTIAFATCGRFPWRQVPAYVSAQVLGSTISIGTLRLLF
GGKHGQFLGTVPAGSDLQSLVLEFIISKTLMFVISGVATDSRAIGELAGLVVGATVVVNVLFAGPISGASMNPARSL
GPAIIANRWEGLWVYIVGPICGTVLGAWAYNLIRFTDRPLLEITNTATASFLKRLTRKDSA

>MaNIP2-5
MASFNEIHDIDVVTVQTLAAEDDFVPAARLRRRKCFQEIFPPFLLRKVIAEVIATFLLVFVTCGAGALNKNNPRVVS
QLGASVAGGLIVTVMIYAVGHISGAHMNPAVTLAFAVSRHFPWIQVPFYWSAQFSGAMIASFILRELLHPITDLGTTT
PSSTPARSLIMEVVVTFSMMFVTSAVATDTKAVGELAGAVGSAVCITSILAGPISGGSMNPARTLGPAVASRKYDAL
WVYFVGPVLGTLSGTMSYRFIRMTEKQPPQSLPSMGSSTAPKSPSTSLKLRRLQSQEMASPAHVNP

Table S2. The full length cDNAs of banana AQPs.

>MaNIP1-1
ATGGAGGAGGGAGCCGCTGGTGATGGGAGAGAAGAGGGCGTCAACCCTGA
CCATGGATATGCGAGCTCTGCCGACAAAGGTTGTGGGTTGAGCTTATCTA
TTCCTTTCTTGCAGAAGATCCTTGCTGAAATATTCGGGACATACTTTCTG
ATCTTCGCGGGATGTGCTTCGGTCACTGTAAATCTGAGCAAGGGAATGAT
CACCTTTCCGGGCATCTGCGTCGTGTGGGGGCTCGCCGTCATGGTCATGG
TGTACTCCGTCGGCCACATATCCGGTGCCCACTTCAACCCGGCCGTCACG
ATCGCCTTCGCCACATGCGGGAGGTTCCCATGGAAACAGGTGCCAGCTTA
CGTTTTTGCTCAGCTTCTGGGCGCGACGCTGGCGAGCGGCACGCTGCGTT
TGATGTTCGGGGGGAAGCACGAGCACTTCCCGGGGACGATACCGGCCGGC
TCGGACGTGCAGTCGCTTGTTCTCGAGTTCATTATCTCGTTCTACCTGAT
GTTTGTGATCTCGGGAGTGGCCACCGACAACAGAGCAATCGGAGAATTGG
CGGGGTTAGCAGTCGGAGCTACAATCTTATTGAATGTGCTCATCGCCGGG
CCTATCTCGGGAGCATCGATGAACCCGGCGAGGACGCTGGGGCCGGCGAT
CGTGGCCAACCGGTGGGAGGGGTTCTGGGTCTACATCGTGGGTCCCATCT
GTGGGACCGTGGTGGGGGCGTGGGCTTACAACCTCATTCGCTTCACCAAC
AAGCCCCTCCGTGAGATCACCAAGAGCGGCTCCTTCCTCAAGAGCTTCAG
GAACAACTCCACCTGA

>MaPIP1-9
ATGATCTTTATCTTGGTCTACTGCACCGCTGGGATCTCAGGTGGCCACAT
CAACCCGGCTGTGACCTTTGGGCTGCTCCTGGCCAGGAAGCTGTCCCTGA
CCAGGGCTCTGTTCTACATGGTGATGCAGTGTCTAGGTGCCATATGCGGT
GCGGGCGTGGTGAAAGGGTTTAAGAAGGGGCTCTATGAGAACAATGGAGG

TGGAGCGAATGTTGTGGCCCCTGGTTACACCAAGGGTGGTGGCTTGGGTG
CTGAGATTGTTGGCACCTTCATCCTGGTTTACACAGTCTTCTCTGCCACT
GATGCCAAGAGGAGTGCTAGGGACTCTCATGTGCCTGTTCTTGCTCCCTT
GCCTATTGGATTTGCAGTGTTCCTTGTTCACCTGGCCACCATCCCCATCA
CCGGCACTGGCATCAATCCTGCCAGAAGCCTTGGAGCTGCAATTGTTTAT
GACAAGAGCCATGCATGGAATGATCATTGGATTTTCTGGGTTGGACCATT
CATTGGAGCTGCTCTTGCTGCTATGTACCAACAGATAGTTATCAGGGCAA
TCCCATTCAAGAGCAGGCCATGA

>MaTIP2-5
ATGGCTTGCATCGCCTTCGGCCGCTGCGATGACTCCTTCAGCGCCACCTC
GCTCAAGGCCTACCTCGCCGAGTTCATCTCCACGCTCCTCTTCGTGTTCG
CCGGCGTCGGCTCTGCCATAGCTTACGGCAAGTTGACGTCGGGCGCGGCC
CTCGACGCGGCGGGGCTCGTGGCGGTGGCCCTCTGTCACGGGCTCGCCCT
CTTCGTCGCCGTCGCGATCGCCGCCAATATCTCCGGCGGCCACGTGAACC
CGGCGGTCACCTTCGGATTGGCTCTCGGGGGGCAGATCACCATCCTCACC
GGACTCCTCTACTGGGTCGCGCAGTTGCTCGGCGCAGTCGTCGGCGCGTT
CCTCCTCAAGTTCGCTACCGGACTCGACACGCCAACCCATAGTTTGGGAG
TGGGAGCCGTGGAGGGAGTGGTGATGGAGATCATCATCACCTTCGCACTC
GTGTACACGGTGTACGCCACCGCCGTCGACCCAAAGAGGGGCTCCCTCGG
CACGGTCGCCCCCATCGCCATCGGCCTTATCGTCGGAGCCAACATCCTCG
CCGCCGGCCCCTTCTCCGGCGGCTCCATGAACCCCGCGCGCTCCTTCGGG
CCGGCAGTGGCGAGCGGCGACTTCGCCGACCTGTGGGTTTACTGGGTCGG
TCCACTTATTGGTGGTGGGCTGGCTGGGCTTGTCTACACCTATGCCTACA
TGTGCACCGACCACACTCCGCTCCCCCAGTAA

>MaTIP2-3
ATGGTGAAGCTCACATTGGGAAGCCTGGGCGACTCTTTCAGCGCGGGGTC
TCTCAAGGCCTATCTTGCTGAGTTCATCGCCACCCTCCTCTTCGTGTTCG
CTGGCGTTGGCTCCGCCATTGCATATGGTAAGTTGACGTCTGGTGCAGCG
CTGGATCCGGCGGGCCTGGTTGCGGTGGCCCTCGCTCATGGCTTGGCCCT
CTTCGTCGGCGTCTCCATGGCGGCCAACATCTCCGGTGGCCACCTTAACC
CGGCTGTCACTTTCGGGCTCGCCGTGGGCGGCCACATCACCCTCCTCACC
GGCGTCTTCTACTGGATCGCCCAGCTCCTCGGCTCCACCGTCGCCTGCCT
CCTCCTCAAGTTCGTCACCGGCGGCATGGCTGTACCGACGCACGGCGTGG
CGGCCGGCATGAGTGAGCTGGAAGGCGTGGTGATGGAGGTGGTCATCACC
TTCGCGCTCGTGTACACGGTGTACGCCACGGCGGCGGACCCGAAGAAGGG
GCCGTTGGGGACGGTGGCGCCCATCGCGATCGGGTTCATCGTCGGGGCCA
ACATCCTGGCAGCCGGGCCCTTCAGCGGCGGCTCCATGAACCCGGCACGG
TCCTTCGGCCCCGCGGTGGCCAGCGGAGACTTCTCCGGCAACTGGGTCTA
CTGGGTGGGGCCGCTGATCGGCGGCGGACTGGCCGGGCTCATCTACGGCG
ACATCTTTATCGGCTCCTACGAGGCGGTCGCGGCGCAGGACTATCCGTAA

>MaTIP4-3

ATGGCCAAGATCGCGCTCGGGAACCACCACGAGGCGGCCGAGCCCGGCTG
CATCCGCGCCGTGCTAGCTGAGGTGGTCCTCACCTTTCTCTTCGTCTTCG
CTGGGGTCGGCGCCGCCATGGCCGCGGAGAAGATGGTGGGCGGGGACTCC
ATCATGGGGCTGACGGCGGTGGCGGTGGCTCACGCGCTGGTGGTGGCGGT
GATGATATCGGCGGGACTCCACATCTCCGGCGGCCACCTGAACCCGGCGG
TGACGCTGGGGCTGGCCGTGGGGGGGCACGTCACCGTCGTCCGGTCGCTG
CTGTACGTGGTGGCCCAGCTGCTGGGTTCCACCCTGGCCTGCCTTCTCCT
CAAATACCTCACTGGTGGACTGGATACTCCGGTGCACACTCTGGCTGCTG
GGATGGGTGCCGTACAAGGAGTGATCATGGAGATAGTGCTCACCTTCTCC
CTGCTCTTCTCCGTCTATGCCACCATGGTGGATCCGAAGAAGGGCATCAT
CGCGGGGCTTGGGCCGCTACTGGTGGGGCTTGTGGTGGGGGCTAACATCC
TCGCCGGCGGCCCGTTCTCGGGCGCGTCAATGAATCCGGCGAGGTCGTTC
GGCCCGGCGTTGGCAGCCTGGAACTGGACCGACCATTGGATCTACTGGGT
CGGACCGCTCGCCGGCGGTGGACTAGCTGGACTCGTCTACGAGCACCTGT
TCATGGTCAGCACCCATGTTCCTCTTCCTAGGGAGGACGAAGGCTTCTGA

>MaNIP2-3

ATGGCTTCCCACGGCACAAGGCCTACTACTACTGCCTGCAACGAAATCCA
TGATGCGGTCACAACTCACACCTCCATCTCTCCCTCTCTTCTCCACCGGA
AGAGCCTCGAAGAACTCTTCCCACCCTTCCTTGCCGAGAAGGTCGTCGCG
GAGACGATCGCCACCTTCCTGCTCGTGTTCGTCACCTGCGGGTCCGCGGC
GTTGAGCAAGAGCGAGGCTGGCGCGGTGTCGCAGCTGGGGGCGTCGGTCG
CCGGTGGGTTGATCGTCACGGTGATGATCTATGCCGTGGGCCACATCTCG
GGGGCGCACATGAACCCCGCCGTCACCTTGGCCTTCGCCGTCTCCCGGCA
TTTCCCATGGATACAGGTTCCATTCTACATCTCTGCTCAGATCTCGGGGG
CCATGGTCTCCTCCTTCGTCCTCCGCGAGCTGCTGCACCCCATCACCGAT
CTCGGTACCACGACGCCGTCGGACACAGCTCTGAAGGCCTTGGTCATGGA
GATCGTGGTCACCTTCTGCATGATGTTCGTCACCTCGGCTGTAGCCACTG
ATTCCAAAGCTGTAGGAGAGTTGGCAGGGTTAGCTGTTGGCTCGGCAGTC
TGCATAACCTCCATTCTAGCTGGGCCGATCTCAGGAGGGTCGATGAACCC
AGCGAGGACGTTAGGCCCGGCGTTGGCGAGCAGGAACTACGATGCTCTAT
GGGTGTATCTTCTTGGGCCTGTGGTCGGCACATTGTTAGGGGCGTTCTCC
TACAGCTTCATAAGGATGACTGAGAAGCAACCGCTGTCGACTACCACCCA
GAAATTGTCCTCCTTCAAGCTTCGGCGTTTGCAGAGCCAGGACATGCCGA
GTCCTTTAGCCGATGCTTCCGGGCGTGTTTAG

>MaTIP2-1

ATGGTGAAGCTCGCATTAGGAAGCTTGGGTGACTCCTTCAGCGTAGTGTC
TCTCAAGTCCTATTTGGCCGAGTTCATTGCCACTCTCCTGTTCGTGTTCG
CTGGCGTCGGCTCCGCCATCGCTTATGGTAAGCTGACGGGTGGTGCAGCG

CTGGACCCGGCGGGCCTGGTCGCCGTGGCCCTCGCGCATGGCTTGGCCCT
CTTCGTCGGTGTCTCCATGGCAGCCAACATCTCCGGCGGCCACCTCAACC
CGGCGGTCACCTTCGGCCTCGCCGTCGGTGGCCACATCACCATCCTCACC
GGCATCTTCTACTGGGTCGCCCAGCTCCTCGGCTCCACCGTCGCTTGCCT
CCTCCTCAAGTTCGTCACCGGCGGATTGGCTGTTCCGACTCACGGCGTGG
CGGCCGGCATGAGCGAGCTGGAGGGGGTGGTGATGGAGGTGGTGATCACC
TTCGCGCTGGTGTACACGGTGTACGCGACGGCGGCGGACCCCAAGAAGGG
GTCGCTGGGGACGGTGGCACCCATCGCGATCGGGTTCATCGTAGGGGCCA
ACATCCTGGCGGCCGGGCCATTCAGCGGCGGTTCCATGAACCCGGCGCGC
TCCTTCGGCCCCGCGGTGGCCAGCGGAGACTTCGCCGGCAACTGGGTCTA
CTGGGTGGGGCCACTCATCGGCGGCGGACTGGCCGGGCTCATCTACGGTG
ACATCTTCATCGGCTCCTACCAGCCCGTAGCAGCTCAGGACTATCCTTGA

>MaPIP2-3
ATGATCTTCATCCTCGTCTACTGCACCGCCGGCATCTCCGGTGGGCACAT
CAACCCCGCGGTGACGTTCGGGCTGTTCCTAGCGCGAAAGGTGTCGTTGG
TGCGCGCCTTCCTCTACATAGTGGCGCAGTGCCTGGGTGCGATCTGTGGC
GTCGGCCTCGTCAAGGGGTTCCAGAAGGCCTACTTCGTCCGCTACGGAGG
CGGCGCCAACGAGCTCAGCGACGGCTACTCCAAGGGCACCGGCCTCGGCG
CCGAGATCATCGGCACCTTCGTCCTCGTCTACACCGTCTTCGCCGCGACC
GACCCCAAGCGCAGTGCTCGCGACTCCCACGTCCCGGTTTTGGCTCCCCT
ACCGATTGGATTCGCGGTTTTCATGGTCCACTTGGCGACGATCCCGATCA
CGGGCACGGGCATCAACCCGGCGAGGAGTTTCGGAGCCGCCGTCATCTAC
AACAAGGACAAGGCCTGGGATGACCAGTGGATCTTCTGGGTGGGGCCGGC
CATCGGTGCTGCCATAGCTGCAGCTTACCACCAATACGTCCTGAGAGCGA
GCGGTGTCAAGGCGTTGGGTTCCTTCAGAAGCAGTGCGTGA

>MaNIP2-4
ATGAAGTGCTTGAAACGATCAGAGATTTTCTCGCAGCCTGGAAGGACACA
GGAAGACCACACCTTGAGGAGGAGTAGTTCTTGGAGATTGGAAATGGCTT
CCCAAACAAGGCCTAACATCTCCAATGAGATCCATGACATAGATGTAGTC
ACAGCTCAGAGCTCCGTCTCTCCCCGTCTTCTTCACCACGAGAGCTTGAG
AGAACTCTTCCCACCCTTCCTTGCAAGAAAGGTCGTTGCTGAGATGATAT
CCACCTTCTTGCTGGTGTTCGTGACCTGCGGTGCCGGCGCCTTGAACAAG
AGCAACTCAGGCGTGGTGTCGCAGCTCGGCGCATCGGTCGCCGGAGGGTT
GATCGTCACGGTGATGATCTATGCCGTCGGCCATATATCAGGGGCACACA
TGAACCCTGCCGTCACCTTCGCCTTCGCCGTCTCGAGGCATTTTCCATGG
ATTCAGGTGCCCTTCTACATGTGTGCTCAGATCTCGGGGGCCATGGTCGC
CTCCTTCGTCCTCCGGGAGCTGTTGCACCCGATCACCAACCTCGGGACGA
CGACGCCGTCTGACACGGCGGCGAAGGCATTGGTCATGGAAACCGTGGTC
ACCTTCTGCATGATGTTCGTCACGTCGGCGGTAGCAACCGATACCAAAGC
TGTAGGAGAGTTGGCAGGGTTAGCTGTTGGTTCATCAGTGTGCATAACCT

CCATTCTAGCTGGGCCGGTCTCAGGAGGATCGATGAACCCCGCGAGGACA
TTAGGACCGGCGGTCGCGAGCAGGAATTACCAATCGCTCTGGGTGTATTT
TGTTGGCCCGGTGCTGGGCACCGTCTCAGGCTCATTCTCCTACAGCTTCA
TTAGAATGACTGAGAAGCAGCAGCATACTACTGCTGCACAGAAGCTGTCC
TCCTTCAAGCTTCGACGCTTGCAGAGCCAGGAAATGGCGAGCCCCACAAG
CAATGCTTTCGAGAATGTGTAG

>MaPIP2-9
ATGATCTTCATCCTCGTCTACTGCACCGCCGGCATCTCCGGTGGGCACAT
CAACCCCGCGGTGACGTTTGGGCTGTTCTTGGCGCGCAAGATTTCGCTCG
TCCGCGCCCTCCTCTACATGATCGCGCAGTGCCTGGGAGCCATCTGCGGC
GTCGGGCTCGTCAAGGGATTCCAGTCCGCCTACTACGTCCGCTACGGTGG
CGGCGCCAACGAGCTCAGCGACGGTTACTCCAAGGGCACCGGCCTGGCCG
CCGAGATCATCGGCACCTTCGTCCTCGTCTACACCGTCTTCTCTGCCACT
GACCCCAAGCGCAATGCCCGCGACTCCCACGTCCCGGTTCTTGCTCCTCT
TCCAATTGGGTTCGCAGTGTTCATGGTCCACTTGGCCACGATTCCGATCA
CCGGCACCGGCATTAACCCGGCGAGGAGCTTCGGAGCGGCCGTCATCTAC
AACAAGGACAAGGCCTGGGATGATCAGTGGATGTTCTGGGTGGGGCCGTT
CATCGGCGCCGCTGTTGCTGCAGCCTACCACCAGTACATCCTTAGAGCCA
GCGGCGCCAAGGCTTTGGGTTCGTCTTCCTCGATCTGA

>MaTIP3-2
ATGCCACCTCGCAGGTTCGCCTTCGGTCGCGCGGAAGATGCCGTTCACCC
GGACACCATGCGTGCGGCACTCTCCGAGTTCATCGCCACGGCTCTCTTTG
TCTTCGCCGCCGAAGGCTCCGTTCTCTCCCTCGGAAAGCTATACAAGGAC
ACCTCCACCGCCGGAGGGCTTGTGGTGGTGGCCATAGCCCACGCTCTGGC
TTTATCTGTCGCGGTCTCCGTCTCCTTGAACATTTCCGGCGGCCATGTCA
ACCCTGCTGTCACACTCGGCGCCCTTGTCGGCGGCCGGATCTCTCTCATA
CTGGCGGTCTTCTACTGGGTGGCTCAGCTACTCGGCGCTGTCGTCGCCGC
TCTCCTCCTCAGGCTGGCAACCGGTGGCATGAGGCCGCTGGGATTCGGGG
TGGCGTCAGGCGTCAGCGAGGGGCACGCAGTCCTGCTGGAGATCGTAATG
ACGTTCGGGCTCGTCTACACCGTGTACGCGACGGCGATCGATCCCAGGAG
AGGCCATCTCGGCATCATCGCGCCTCTCGCCATCGGCTTCATCCTGGGCG
CCAACATCCTTGCCGGCGGGCCATTCGACGGCGCCGCGATGAATCCCGCG
CGGGCCTTCGGACCGGCAGTCGTCGGCTGGAGGTGGAAGAGCCACTGGGT
GTACTGGGTTGGCCCCTTGGTAGGAGCAGCTCTTGCAGGGCTTATCTATG
AGTTCCTTGTGATCCCGGATGAGACTCCCCGCACTCACCAGCCCTTGGCT
CCCGAGGACTACTAA

>MaTIP2-4
ATGGCTGGAATCGCCTTCGGCCGCTTCGATGACTCTTTCAGTGTCGGCTC
ACTGAAGGCCTACCTCGCCGAGTTCATCTCCACTCTCCTCTTCGTCTTCG

CTGGCGTCGGATCAGCTATAGCTTACAACAAGTTGACGTCGAGCGCAGCT
CTGGATCCTGCGGGGCTCGTCGCCATCGCCGTCTGCCATGGGTTTGCGCT
CTTCGTGGCGGTATCGGTGGGCTTCAACATCTCCGGCGGCCACGTGAATC
CGGCGGTCACCTTCGGGTTGGCTCTTGGAGGGCAGATCACCATCCTCACT
GGCATCTTATACTGGATCTCCCAGCTGCTTGGAGCTGTTGTCGGAGCTTT
CCTCCTCAAGTTCTCGACCGGACTGGACACCCCCACTCATGGACTGGGAG
CAGGAGTGGGAGCAGGAGAAGGGGTGGTGATGGAGATCATCATCACCTTC
GCCCTGGTGTACACGGTGTACGCGACGGCAGCCGACCCGAAGAAGGGCTC
CCTCGGCACCATCGCCCCGATCGCCATCGGCTTCATCGTCGGCGCCAACA
TCCTCGCCGCCGGCCCCTTCTCCGGCGGGTCGATGAACCCGGCTCGCTCC
TTCGGGCCCGCCGTCGCTAGCGGGGACTTCTCCGACCTCTGGATCTACTT
TGTCGGCCCCCTGATTGGCGGCGGCCTCGCAGGGTTGGTGTACACCTACG
CCTACTTGTTACACGACCACCAGCCACTTCCGCAGTGA

>MaPIP1-1
ATGATCTTTGCTTTGGTCTACTGCACTGCTGGCATCTCCGGTGGCCATAT
CAACCCTGCTGTGACGTTTGGGCTGTTCCTGGCGCGGAAGCTGTCCCTCA
CAAGGGCTGTCTTCTACATGGTGATGCAGTGCCTGGGTGCCGTCTGCGGA
GCTGGTGTGGTGAAGGGGTTCCAGAAGGGCGTCTATGAAAGCAACGGTGG
CGGAGCCAACGTGGTGGCCTCTGGCTACTCCAAGGGTGACGGCCTGGGTG
CTGAGATTGTCGGCACCTTCATCCTTGTCTACACAGTCTTCTCTGCTACC
GACGCCAAGCGGAATGCCAGGGACTCCCATGTGCCTCTCCTTGCTCCCTT
GCCTATTGGATTTGCTGTTTTCCTGGTTCACCTGGCAACCATCCCCATCA
CCGGCACTGGCATCAACCCAGCTCGGAGCCTGGGAGCTGCCATCATCTAC
AACAAGGACCATGCCTGGGATGACCATTGGATCTTCTGGGTTGGTCCCTT
CATTGGAGCTGCCCTTGCTGCCATCTACCACCAGGTAGTCATCAGAGCCA
TCCCCTTCAAGAACAGATCCTGA

>MaPIP1-4
ATGATCTTTGCTTTGGTCTACTGCACCGCCGGCATCTCCGGTGGCCATAT
CAACCCTGCTGTGACCTTTGGGCTGTTCCTGGCGAGGAAGCTGTCCCTCA
CACGGGCCATCTTCTACATGGTGATGCAGTGCCTGGGCGCCATCTGCGGT
GCCGGCGTCGTCAAGGGGTTCCAAAAGGGAGTCTACCAGAGCAACGGTGG
CGGAGCCAACGTCGTGGCCTCCGGCTACTCCAAGGGCGACGGATTGGGCG
CCGAGATCGTGGGCACCTTCATCCTGGTCTACACCGTCTTCTCCGCGACC
GACGCCAAGCGCAACGCCAGGGACTCCCACGTGCCTATTCTTGCGCCCTT
GCCCATCGGATTCGCTGTCTTCCTGGTTCACCTGGCAACCATCCCGATCA
CCGGCACCGGCATCAACCCTGCTCGGAGCCTGGGAGCTGCCGTCATCTAC
AACAAGGACCATGCCTGGGATGACCATTGGATCTTCTGGGTCGGTCCCTT
CATTGGAGCTGCCCTCGCTGCCCTCTACCACCAGGTGGTCATCAGAGCCA
TCCCATTCAAGAGCAGATCCTGA

>MaSIP2-2
ATGGGGCGGCTGGGGCTCGTGATGTGCGACGCGGCTATGTCGTTCATGTG
GGTGTGGGCCGGCGCGCTCGTCAAGCTCCTGGTCTACGACGCCCTCGGCC
TGGGCCACCGCCCCGGCGGCGAGGCCCTCAAGATGGCCCTCGTTGTCGGC
TACATGTTCCTCTTCGCGTGGCTCGGCCACGTCACGCGGGGTGGCGCCTA
CAACCCTCTCACCGTGCTCTCCTACGCCTTCTCCGGTGGTCCCGAGGGGT
TCCTTTTCACAGCCCTGGGAAGGATCCCTGCGCAGGTGATAGGATCAGCA
ACTGGTGTTAGATTCATCAAACAAACCTTTCCTTCGATAGGCCATGGACC
TCGTTTGAGCATTGATATCCATCGTGGGGCATGGACAGAAGGGTTCCTTA
CGTTTATGATCGTGATGGCCTCACTAATGCTAAAGAAAAAAGATCCTGGA
AGTTTCTTCATGAAAACATGGATTTCAAGCATTTTCAAGTTGGCACTTAA
TGTCCTTGGTTCTGATTTAACTGGAGGAATTATGAACCCTGCCTCTGCCT
TTGCTTGGGCATATGCTCGGGGAGATCACATAACGTTGGATCAACTAATT
GTTTACTGGTTTGCGCCCATTCAAGCAACTTTATTAGCAGTCTGGACATT
CGGATTGCTTACCGAGCCAAAGAGCAAGGTGCAAAAAGCTGAAGAAAACA
AGGTTAAGTCGGAGTGA

>MaPIP2-13
ATGATATTTGTCCTCGTCTACTGCACCGCCGGCATCTCTGGTGGGCACAT
AAACCCGGCCGTAACATTCGGCCTGTTTCTGGCGCGGAAGGTGTCGCTGC
TGCGCGCGGTGTTCTACATGGTGGCGCAGTGCATGGGGGCCATATGCGGC
GTCGGAATCGTGAAGGGCATCATGAAACACCAGTTCAACAGGTTCGGCGG
CGGGGCTAATGTGGTGGCTCCAGGCTACTCCAAGGGCACCGCGCTCGGCG
CCGAGATCATCGGCACCTTCTTGCTCGTCTACACCGTCTTCGCCGCCACC
GACCCCAAGCGCCGCGCTCGCGACTCCCACGTCCCGGTGTTGGCTCCTCT
GCCCATCGGGTTTGCCGTGTTCATGGTTCACTTGGCCACCATTCCCATAA
CTGGCACGGGCATCAACCCCGCTAGGAGCTTCGGACCCGCCGTCATTTAC
AACCGGCACAAGCCCTGGCATGATCACTGGATCTTCTGGGTGGGTCCGTT
CGTCGGAGCACTGGCGGCGGAGGTATACCACCAGCACGTGTTGAGGGCGG
CGAACGTCAAGACTTTGGGCTCGTTCAGGAGCAGCCGGAGCAACTGTTAG

>MaTIP5-1
ATGTCAAGTAAACTCCTGTGCTTTTTTACTTCTGGAGTCAATTTTTGGCT
GTCTGCCGCATCTCTCCGTTCCTATCTCGCCGAGTTCATCTCCACCTTCT
TCTTCGTCTTCGCCGCCGTCGGCTCCGCCATCTCTGCCCGGATGTTAACG
CCGGATGTCACGTCGGACGCGTCGTCGCTGGTCGCGACGGCGCTCGCGCA
GGGCTTCGCGCTGTTCGCTGCGGTCTACATCGCCGCCGACATCTCGGGGG
GCCACGTAAACCCCGCCGTCACCTTCGGCCTCGCCGTCGCGGGGCACATC
GGCGTCCCGACTGCCATCTTTATTGGATCTCCCAGTTGGGTGGCTCCAT
TCTCGCTTGCCTCCTCCTCCGCGTCGCCTCGGCCGGACAGGCGATTCCGA
CGACGGGGATAGGGACGGAGATGACGGGCTTCGGCGGGGCGGTGGTGGAG
AGCGCGATCACGTTCATTCTGGTGTACACGGTCTACGTGGCTGCGGACCC

CGGGGGCGGCGGCGGAGACGGAAAGAGGAAGAGGAAAATGAGGCGAGAGG
TGGCGGGGCCCCTCGCGGTAGGTCTGACGGCAGGGGCGTGCGTCCTGGCG
GCCGCCTCCCTCACGGGGGGATCCATGAACCCGGCGAGGTCCTTCGGCCC
GGCCGTCGTCAGCGGCAACTTCAAGAACCACGCGGTCTACTGGGTCGGGC
CCCTCATCGGCGCAGCCCTGGCGGCGCTGGTTCACCAATACCTGGTGTTC
CCTTCCGCCTCCTCCGACGCCTATCCCAACTCCACGGTGTAG

>MaTIP2-2
ATGGCCGGCATCGCCTTTGGGCAATTTGACGACTCTTTCAGTGTCGGCAC
ACTCAAAGCCTACCTCGCCGAGTTCATCTCCACTCTTCTCTTCGTCTTCG
CCGGCGTCGGATCAGCTATCGCTTACAACAAGCTGACGTCGAGCGCAGCT
TTGGATCCTGCCGGGCTGGTCGCGATCGCTGTTTGCCATGGTTTAGCGCT
CTTCGTGGCGGTGTCGGTCGGCGCCAATATCTCCGGCGGCCACGTGAATC
CGGCGGTCACCTTCGGGCTGGCTCTGGGAGGCCAGATTACCATCCTCACT
GGGATCTTCTACTGGGTCGCGCAGTTGCTTGGAGCTGTCGTTGGAGCTTT
TCTCGTCAAGTTCGCGACAGGACTGGACACCCCCACCCATGGCCTGGGAG
ACGGAGTGGGAGCAGGAGAGGCGGTGGTGATGGAGATCATCATCACCTTC
GCCCTCGTGTACACGGTGTACGCGACGGCGGCCGACCCGAAGAAGGGCTC
TCTCGGCACCATCGCCCCCATAGCCATCGGCTTCATCGTCGGCGCCAACA
TCCTCGCGGCGGGCCCCTTCTCCGGAGGGTCGATGAACCCGGCGCGCTCC
TTCGGGCCGGCCGTCGCCAGCGGGAACTTCTCCGACCTCTGGATCTACTG
GGTCGGTCCCCTCATCGGTGGCGGCATCGCCGGGCTGGTGTACACCTACG
CCTACATGTGCTCAGACCACCAGCCACTCCCTCAGTGA

>MaPIP1-7
ATGATCTTTGCCCTCGTCTACTGCACCGCTGGGATCTCAGGTGGCCACAT
CAACCCGGCCGTGACCTTCGGGCTGCTCCTGGCGAGGAAGCTCTCCCTGA
ACCGAGCTCTCTTCTACATGGTGATGCAGTGCCTGGGTGCCATCTGCGGT
GCTGGTGTGGTGAAGGGGTTTCAGAAGGGGCTCTACCAGAGCAATGGTGG
CGGAGCAAACGTTGTGGCCGCTGGCTACACCAAGGGTGATGGCCTGGGTG
CTGAGATTGTTGGCACCTTCATCCTTGTCTACACTGTCTTCTCTGCTACT
GATGCCAAGAGGAACGCTAGGGACTCTCATGTGCCTATCCTCGCCCCATT
ACCTATTGGTTTTGCCGTATTTCTTGTTCATCTGGCCACCATCCCCATCA
CCGGCACTGGCATCAACCCTGCAAGAAGCTTTGGAGCTGCAGTTATCTAC
AACAAGGACCATGCTTGGGATGACCATTGGATCTTCTGGGTTGGACCATT
CATCGGAGCTGCTCTTGCTGCCCTCTACCACCAGGTGGTGATCAGGGCAA
TCCCATTCAAGAACAGGACCTGA

>MaPIP2-4
ATGATCTTTATTCTCGTCTACTGCACCGCCGGCATCTCCGGTGGGCACAT
CAACCCGGCGGTGACGCTGGGGCTATTCTTGGCGCGCAAGGTCTCGCTGG
TCCGCGCCCTCCTCTACATGGTGGCGCAGTGCCTGGGAGCCATATGCGGA

GTCGGCCTCGTCAAGGGATTCCAAGAGGCCTACTTCGTCCGCTACGGTGG
CGGCGCCAACGAGCTCAGCGCCGGCTACTCCAAGGGCACCGGCCTCGCCG
CCGAGATCATCGGTACCTTCGTCCTCGTCTACACCGTCTTCGCCGCCACC
GATCCGAAGCGCAACGCCCGTGATTCCCACGTGCCGGTTTTGGCTCCTCT
TCCAATTGGGTTCGCAGTTTTCATGGTGCACTTGGCCACGATCCCGATCA
CCGGCACCGGCATCAACCCTGCGAGGAGCTTGGGAGCTGCCGTCATCTAC
AACCAGGACAAGGCCTGGGATGACCAGTGGATCTTCTGGGTGGGGCCTTT
CGTCGGTGCTGCCATTGCTGCAGCCTATCACCAGTACGTCCTGAGAGCGA
GCGGTGCCAAAGCTATGGGGTCCTTCGGGAGCAATGCATGA

>MaTIP4-1
ATGAGAAAGATAACGTTAGGCAGCAGAAACGAGGCGGTTGAGCCGGACTT
TGTCCGTTCGGTCTTCACCGAGCTGCTCCTCACCTTCCTCTTTGTCTTCG
CCGGGGTCGGCGCCGTCATGACTGCAGAGGAGGTCGCAGGAGGCGAGGAC
CGGATCATGTGGGTGGTGGCGGCCCCGGCAGCAGCTCAGGCGATGCTGGT
GGCGATGATTACAGCGGTTGGCCTCGATGTCTCGGCCGGCCACCTGAACC
CCGCCGTCACCATCGGGTTTGCGGCCGGCGGCTACGTCACCGTCGTCCGC
TGCGTCCTCTACGTGATCGCCCAGCTATTGGGCTCCTCCATGGCCTGCCT
TCTCCTCAAGTACGTTGCTGCAGGACTGGATGTACTCCCAGTTCATGCAC
TTGCTGCAGGAATGGATCCGCTCCAAGGCGTGATCATGGAGGCCGTCTTC
ACCTTCTCCATGGTCTTCGCCATCTACGCCTTGATCATGGACCCCAAGAA
GGGCGCAATCGCCGGCTCCGCTCCCCTCCTCATCGGCCTCACCGTCGGCG
CCAACTCCTTGGCCGGCGGGGCCTTCTCCGGCGCGTCGATGAACCCGGCG
AGGTCATTCGGGCCGGCGCTGGCCAACTGGGACTGGACCAATCACTGGGT
GTATTGGCTGGGGCCCCTCGTCGGAAGTGGCCTCGCAGGGTTCGCTCACC
ACCACCTCTACGTCGCCGGGACGCATGGCGTTCTCCTTCCCAAAGATGAC
GAAGTCGGCTTTTGA

>MaPIP2-12
ATGATCTTTGTCCTCGTCTACTGCACCGCCGACATCTCTGGCGGGCACAT
AAACCCGGCTGTGACGTTGGGTCTATTCGTGGCGCGGAAGGTGTCGCTGC
TGCGCGCAGTGCTGTACATGGTGGCGCAGTGCCTGGGCGCCATATGCGGC
GTCCGGATCGTGAAGGCCATCATGAAGCAGCAATTCAACGCTTTCGGCGG
CGGGGTTAATGTGGTGGCCCCAGGCCACTCCAAGGGCACCGCCCTCGGCA
CCGAGATCGTCGGCACCTTCGTCCTCGTCTACACCGTCTTCTCCGCCACT
GACCCCAAACGCAGCGCCCGCGACTCTCACGTTCCGGTGTTGGCTCCTCT
GTCGATCGGGTTTGCGGTGTTCATGGTACACTTGGCGACGATTCCGATCA
CGGGCACCGGCATCAACCCGGCAAGGAGCTTAGGAGCTGCGGTCATTTTC
AATCAGCACAAGCCGTGGCATGATCACTGGATCTTCTGGGTGGGTCCTTT
CGGAGGAGCTCTGGCGGCGGCGGTGTATTATCAGTACGTGTTGAGGGCGT
CGACTATTAAGGATTTGGTCTCCTTCAGGAGCAGCCGCAGCAACTAA

>MaTIP4-2
ATGGCGAGGATAAAGCTGGGAAGCAGAAAGGAGATGACCGACCCGGAGTT
CGCTCGGTCCGTCCTCACCGAGCTGCTCCTCACCTTCCTTTTCGTCTTCG
TCGGCGTCGCTGCGTCCATGACCGCCGGGAAGATGGCAGGAGGGCAGGAC
TCGATCATGGGACTGACGGCGGTGGCGGTGGCTCAGGCGATGCTGGTGGC
GGTGATGGTAGCCGTCGGCCTCGACGTCTCGGCTGGGCACTTGAACCCCG
CCGTCACGATCGGGTTTGCCGCCGGCGGCTACGTCACCGTCTTCCGCTGC
GTCCTCTACGTGATCGTCCAGCTGCTGGGCTCTTCCATGGCGTGCCTCCT
CCTCCAGTACATCGCTGGAGGGCAGGCCGTCCCAGTTCACGCGCTCGGTG
TGGGCATAGGGCCCCTTCAAGGTGCGATCATGGAGGTCGTCCTCACCTTC
TCCATGGTCTTCTCCATCTACGCCATCATCGTAGACCCCAAGAAGGGCAT
CGTGTCGGTGCTGGCGCCGCTGCTCATTGGTCTCATCGTGGGGGCCAACA
CGCTCGCCGGCGGGCCCTTCTCGGGGGCTTCCATGAACCCGGCGAGGTCC
TTCGGGCCCGCCCTGGCCACCTGGGACTGGACCAATCACTGGGTGTACTG
GGCGGGGCCCTTCGTGGGGAGTGGCCTCGCGGGGTTCGTCTACGACCACC
TCTATCTCATGCGGCCTCGTGACGACCTCCCTGGGGATGAAGAAAGCATC
ACCAAGCCACTCTGTTAG

>MaSIP2-1
ATGCTTGACCAGAAAGAGTTTGTAAATAAAAAGGAAACAGATGTAGAAGA
AGAGAAGGAACACAGCAACCAAGCGTCTCGGGTTAAGCTCATTGTCTCCG
ACTCCTTCCTCTCCTTCATGTGGGTGCTGTCAGGATCCGTTATTAGGTAT
TTGATTTACATGATTCTAGGGACTGGAATGGACCCAATTTCTGTTCTATT
AAAAGGATATTTGGCCCTGGTGTACCTCTACTACTTCTCACAGCTTCGAA
AGGTGACCAATGGTGGAACATATAACCCTCTCTTTGTCCTCTGCCATGCC
ATATCTGATAATTTTGTCGAGTTCCTTTATGCAGTGTTTGGAAGGATTCC
TGCTCAGGTTCTTGGATCTGTCATTGGGGTTTGGTTAATTAATGCAACCT
TCCCTGCAGCGGCTAATGGACCTCGTTTGAATGTTGATGTTAGTTATGGA
GCATTGATTGAAGGACTCATTACATTTGCGATTATCATTGTCTCTCTAGG
GCTAAACAAATTCCCAAGATCTTCCCATAAGACATGGATATCAAGTTTTG
CCAAACTTGCACTTCACGTCCTTGCTTCTGACATCACCGGTGGAGTTATG
AACCCTGCTTCTGCCTTTGGATGGGCATATGCTCAAGGGAAGCATCTAAC
GAAGGAGCATTTGTGTGTGTACTGGCTTGCACCTCTGGAGGCAACTTTAT
TGGTTGTATGGATTTGCAGCTTGTTCATTAAGCTGCCGAAGCGAAAGAGG
CAGCATGAGATGCAATACAAGGATAAACTAGTTTGA

>MaPIP1-5
ATGGGCGTGGTCAAGTCCGACACCAAGTGCTCCACGGTGGGCATCCAAGG
CATCGCCTGGGCCTTCGGTGGCATGATCTTCGCCTTGGTCTACTGTACCG
CTGGGATCTCAGGTGGACATATCAACCCGGCGGTGACCTTCGGGCTGTTC
CTGGCAAGGAAGCTCTCCCTCACCAGGGCGCTGTTCTACATGGTGATGCA
GTGCCTGGGTGCCATCTGCGGTGCTGGTGTGGTGAAGGGGTTTCGGAAGG

GGCTCTACGAGAGCAACGGCGGTGGAGCAAACGTGGTGGCCGCTGGCTAC
ACCAAGGGTGATGGCTTGGGTGCTGAGATTGTGGGCACCTTCATTCTTGT
CTACACCGTCTTCTCTGCCACCGATGCCAAGAGGAATGCTAGGGACTCTC
ATGTGCCCATACTCGCCCCCTTGCCCATTGGTTTTGCAGTTTTCCTGGTT
CACTTGGCCACCATCCCCATTACCGGCACTGGCATCAATCCCGCAAGAAG
CCTTGGAGCTGCCGTCATCTACAATAAAGACCATGCTTGGGATGACCACT
GGATCTTCTGGGTTGGACCATTCATCGGAGCTGCTCTTGCTGCCATGTAC
CACCAGGTTGTGATCAGGGCAATCCCATTCAAGAGCAGGCCCTGA

>MaTIP1-6
ATGCCGATCCCTCGAATCGCCGTTGGAACTCAGGAGGAGGCGACTCACCC
GGGCACGCTCAAGGCCGCCCTCGCCGAGTTCATCTCCACCCTCATCTTCG
TTTTCGCTGGCCAAGGCTCCGGCATGGCCTTTAGCAAGCTGACGGGCGGC
GCAGCCACCACTCCGGCCGGCCTCATAGCGGCAGCCCTAGCGCACGCCTT
CGCCCTATTCGTGGCCGTGTCCGTGGGCGCCAACATCTCTGGTGGCCACG
TGAATCCGGCCGTAACCTTCGGCGTCTTCATCGGCGGCAACATCACCCTC
CTCCGCAGCATCATATACTGGATCGCACAGCTCCTCGGCTCCACCGTCGC
GTGCCTCCTGCTCCGCTACTCCACCGGCGGCCTGTCCACGGGCAGCTTCG
CTCTCTCCGGCGTCAGCGTGTGGGAGGCGCTCGTGCTGGAGATCGTCATG
ACCTTCGGCCTCGTGTACACCGTGTACGCGACGGCGGTGGACCCCAAGAA
GGGTAGCCTGGGCACCATTGCGCCCATCGCCATCGGCTTCATCGTGGGCG
CCAACATCCTGGCCGGCGGGGCCTTCGACGGCGCGTCCATGAACCCGGCC
GTGTCCTTCGGCCCCGCCCTGGTGAGCTGGTCCTGGGACGACCACTGGGT
CTACTGGGCCGGACCCCTCATCGGCGGCGGCCTCGCCGGTCTCGTCTACG
AGTTTTTCTTCATCTCCCACACTCACGAGCAACTCTCCTCCGCCGACTAC
TGA

>MaPIP2-5
ATGATATTCATCCTCGTCTACTGCACCGCCGGCATCTCCGGTGGCCACAT
AAACCCGGCGGTGACGTTTGGGCTGTTCCTGGCACGAAAGGTCTCCCTCA
TCCGCGCGCTGCTCTACATGATCGGCCAGTGCTTGGGAGCGATCTGTGGG
GTTGGGCTGGTGAAGGGATTCCAGAAGGCCTTCTTCGTCCGCTACGGCGG
CGGCGCCAACGAGCTCAGCGACGGCTACTCCAAGGGCACCGGCCTTGCCG
CAGAGATCATCGGCACCTTCGTCCTCGTCTACACCGTATTCTCCGCCACC
GACCCCAAGCGCAGCGCCCGCGACTCCCATGTGCCGGTTCTGGCGCCACT
CCCGATCGGGTTCGCAGTTTTCATGGTTCACCTCGCCACGATTCCCATCA
CCGGCACTGGCATCAACCCGGCGAGGAGCTTTGGAGCTGCTGTCATCTAC
AACAAGGACAAGGCTTGGGACGACCAGTGGATATTCTGGGTGGGGCCTCT
CATCGGTGCCGCCATTGCCGCAGCTTATCACCAATACATCCTAAGAGCCG
GAGCTGTCAAAGCTTTGGGTTCTTTCCGGAGCAATGCATGA

>MaNIP2-2

ATGGCTTCCTCCACAAGGCCTAATAGCTCCAATGAAATCCATGACATCGA
TGTAGTTACAGCTCAGAACTCCTACATCTCTCCGACTCTTCTTCACCAGA
AGAGCCTTAAAGAAGTCTTCCCACCCTTTCTTGCAAGAAAGGTTGTAGCT
GAGACGATCGCTACCTTTTTACTGGTATTCGCCACCTGCGGCTCCGCCGC
GTTGAGCAAGAGCAACCCAGGCCTGGTCTCGCAGCTCGGGGCATCAGTCG
CCGGAGGACTGATCGTGACGGTGATGATTTACGCCGTGGGGCACATCTCG
GGCGCCCACATGAACCCTGCCGTCACGTTGGCCTTCGCCGTCGCCAGGCA
TTTTCCATGGATTCAGGTCCCCTTTTACATGGCTGCTCAGATCTCCGGCG
CCATGATCGCTTCCTTCGTCCTCCGCGAGCTGCTGCACCCGATCACCGAT
CTCGGGACCACGGCGCCGTCGGATACGGCGGTGAAGGCACTGGTCATGGA
AATCGTGGTGACCTTCTGCATGATGTTCGTCACGTCAGCGGTGGCGACGG
ACACCAAAGCTGTAGGAGAGTTGGCAGGGTTAGCTGTGGGTTCATCGGTG
TGCATAACCTCCATTCTAGCTGGGCCGATATCTGGAGGATCGATGAACCC
GGCGAGGACACTCGGACCGGCCGTCGCGAGCAGCAATTACGACTCGCTTT
GGGTGTATTTTCTGGGCCCGGTGTTGGGCACCTTGTCAGGAGCATGCTCC
TACAGTTTCATAAGGATGACCGAGACGCAACCACAAGCCACTGCTGCACA
GAAGCTATCCTCCTTCAAGCTCCGCCGCTTGCAGAGCCTGGAAATGGCGA
GTCCTACCAACAACGCTTTCGATAACATTTAG

>MaTIP1-4
ATGCCGATCCTTCGCATAACGATCGGAACGCCAGAGGAGGCGCGCCACCC
TACCGCGCTTAAAGCCGCTCTCGCCGAGTTCATCTCCGTGCTCATTTTCG
TCTTCGCCGGCCAGGGATCGGGGATGGCGTTCAATAAGCTCACGGATGAT
GGCTCCACAACCCCAGCGGGGTTGGTGTCGGCGTCCTTGGCCCATGGCTT
CGGCCTCTACGTCGCAGTAGCCGTCGGGGCCAACATCTCCGGCGGCCACG
TCAACCCGGCCGTCACCTTCGGCGCCTTCCTCGGTGGCAACATCACGCTG
CTTCGGGGCATTTTGTACTGGATCGCGCAGCTGCTCGGCTCCGTGGTCGC
CTGCCTGCTTCTCAAGTTCGCCACCGGCGGCCTGGAAACAACGCCCTTCT
CGCTGTCGAGCAGCGTGACCGTGTGGAACGCGTTGGTGTTTGAGATCGTG
ATGACCTTCGGGCTGGTGTACACGGTCTACGCGACGGCCATAGATCCGAA
GAAGGGCAACCTGGGAATCATCGCGCCCCTCGCGATCGGGCTCGTCGTGG
GTGCCAACATCCTGGCAGGCGGGGCGTTCGACGGCGCCTCCATGAACCCG
GCCGTCTCCTTCGGCCCCGCGGTGGTCAGCTGGACCTGGGACAATCACTG
GGTATACTGGGTCGGCCCGTTGCTCGGCGGGGGCATCGCGGCCTTGGTCT
ACGACGGCGTCTTCATCGGCTTCGGCACCCATGAGCAGCTCCCTACCACA
GACTACTAG

>MaPIP2-10
ATGATCTTCATCCTCGTCTACTGCACCGCCGGCATCTCTGGAGGGCACAT
CAACCCCGCGGTCACGCTCGGGCTGTTCTTGGCGCGGAAGGTGTCGCTGA
TACGGGCGTTGCTGTACATGGTGGCGCAGTGCTTGGGAGCCATCGTCGGG
GTGGGGATCGTGAAGGGGATCATGAAGCACCAGTATAACTCCCTCGGCGG

CGGAGCCAACATGGTCGCCGCCGGCTACTCCAAGGGTACCGCCCTCGGGG
CCGAGATCATCGGCACCTTCGTCCTCGTCTACACCGTCTTCTCTGCCACC
GACCCCAAGCGCAGCGCTCGCGACTCGCACGTCCCCGTGTTGGCACCACT
CCCCATCGGGTTCGCCGTGTTCATGGTGCACCTCGCCACTATCCCCATCA
CCGGGACCGGCATCAACCCTGCTCGGAGCCTTGGCGCTGCAGTCATCTAC
AACCAGGACAAGCCCTGGGATGACCATTGGATCTTCTGGGTGGGTCCCTT
CGTCGGAGCGTTAGCCGCGGCGGCGTACCACCAGTACATCCTGAGAGCAG
CGGCCATCAAGGCTCTGGGTTCCTTCAGGAGCAACCCCACCAACTAA

>MaNIP3-2
ATGCATCAATATCTAATTCAGCAAATATTGACCAAAAATACACTGAAACC
GAAGCCTTTGGCAATGCCGGAGCCGGAGACGCCGAACGTGTCAGCCCCGG
CGACGCCGGGCACGCCGGGCGCGCCGCTCTTCAACTCGCTCCGAGTTGAC
TCGCTGTCTTACGACCGGAAGTCGATGCCGAGGTGCAACAGGTGCCTCCC
GTTGGAGTCGTGGGCCTCCTCCCCCCACACGTGCTTCATCGAGCTCCCCA
AGCCCGACGTCTCCCTCACCCGCAAGCTGGGAGCAGAATTTGTGGGGACA
TTCATCCTCATATTTGGCGCCACGGCGGCACCGATCGTGAACCAGAAGTA
CAACGGCGCCGAGACCCTCATCGGCAACGCCGCCTGCGCCGGCCTGGCGG
TCATGATCGTCATCCTGTCCACAGGCCACATTTCCGGCGCCCACCTCAAC
CCGTCCCTCACCATCGCCTTCGCGATGCTGCGCCACTTCCCCTGGGCCCA
CGTCCCCGCCTACATCCTCGCCCAGGTCTCCGCCTCCATCTGCGCCTCCT
TCGCCCTCAAGGCCGTCTTCCACCCCTTCCTCTCCGGCGGCGTCACCGTG
CCCTCCGTCAGCTCCCCCCAGGCCTTCTTCATCGAGTTCCTCATCACCTT
CAACCTCCTCTTCGTCGTCACCGCCGTCGCCACCGACACTCGTGCCGTGG
GGGAATTGGCTGGAATCGCGGTGGGGGCCACGGTCATGCTCAACATTCTG
GTGGCCGGGCCATCGAGCGGGGGATCGATGAACCCGGTTCGGACGCTTGG
GCCGGCGGTAGCGGCCGGCAATTACGAGCGGATTTGGATATATCTGGTGG
CGCCGACGGCCGGGGCAGTCACGGGAGCCGCCGTTTACACGGCCGTCAAG
CTCAAGGAGGAGGACGGCGAGATGCCACGGCAACAACTTCTGGAAGATTA
TCCACGTCATACTAGATATGCTTTGCAAAATGATTCTTGGATTGTGGCCT
CTTATTTTTATCTTTGTTCTGTTAAACATATATTTTTTTATTGCAGATG
TTGAATTGTGAATATTGTAAGCTACGATAG

>MaTIP3-1
ATGCCGCCCCGCAGGTTCGCCTTCGGCCGCACCGACGATGCCGTCCACCC
GGATACCATGCGTGCCGCTCTCTCGGAGTTCATCGCCACCGCTCTCTTCG
TCTTCGCCGCCGAGGGCTCCATTCTCTCTCTCGGAAAGCTCTACAAGGAC
ACCTCCACCGCAGGGGGGCTGGTGGTGGTGGCTATAGCCCACGCGCTAGC
TTTGGCTGTAGCCGTGGCCATCGCCTTTAACATATCGGGTGGCCACGTCA
ATCCTGCGGTCACGCTCGGCGCTCTCGTCGGCGGCCGGATCTCCCTTGTG
CGGGCGGTATTCTACTGGGTGGCGCAGCTACTCGGCGCCGTCGTAGCCGC
CCTCCTCCTCAGGCTCGCGACCGGTGGCATGAGGCCGGTTGGATTCTCGG

TGGCGTCGGGCGTCAGTGATTGGCACGCCGTCCTGCTGGAGATAGTGATG
ACGTTCGGGCTGGTCTACACCGTCTACGCGACTGCGATCGACCCCAAGAG
GGGGCACCTCGGCACCATCGCGCCTCTGGCCATTGGCTTCATCCTGGGCG
CCAACATCCTTGCCGGGGGACCGTTCGACGGCGCGGCAATGAACCCGGCG
AGGGCCTTCGGACCGGCACTTATCGGGTGGAGGTGGAAGCACCACTGGGT
GTACTGGGTTGGCCCCTTCATAGGTGCAGCACTTGCAGGCGTTATGTATG
AGTTCCTTATGATCCCAGCTGAGGCTCCTCGCACTCACCAGCCTTTGGCT
CCTGAGGACTATTAG

>MaPIP1-6
ATGGGGGTGGTCAAGTCCAGCACCAAGTGCTCGACGGTGGGCATCCAGGG
CATCGCCTGGGCCTTTGGCGGCATGATCTTTGCGCTGGTCTATTGCACCG
CTGGGATCTCAGGTGGCCATATCAACCCGGCGGTGACCTTCGGGTTGTTC
CTGGCGAGGAAGCTCTCCCTCACCAGGGCTCTGTTCTACATGGTGATGCA
GTGCCTGGGTGCCATCTGCGGGGCTGGTGTGGTCAAGGGGTTCCAAAAGG
GGCTCTATGAGAACAACGGTGGTGGGGCAAACGTGGTGGCTCCTGGCTAC
ACCAAGGGCGATGGCTTGGGTGCTGAGATCGTGGGCACCTTCATCCTCGT
CTACACTGTCTTCTCTGCCACTGATGCCAAGAGGAGTGCTCGGGACTCTC
ATGTGCCTATCCTTGCTCCGCTGCCTATTGGCTTTGCAGTGTTCCTGGTT
CACCTGGCTACCATCCCCATCACTGGCACTGGTATCAATCCTGCCAGGAG
TCTTGGAGCTGCAATCATCTACAACAAGGGCCATGCTTGGGATGACCATT
GGATATTCTGGGTTGGGCCATTCATTGGAGCTGCTCTTGCTGCCTTGTAC
CACCAGGTAGTCATCAGGGCAATCCCATTCAAGAGCAGGTCATGA

>MaTIP1-3
ATGCCGATCACTCAGATAGCCATCGGGACTACGGCCGAGGCAACCCACCC
GACTGCACTCAAGGCCGCGCTCGCCGAGTTCATATGCACCTTCATCTTCG
TCTTCGCCGGCCAAGGCTCCGGCATGGCCTACAACAAGTTGACGAGCGAC
GGGGCTGCGACGCCCGAGGGGCTGATCGCGGCGGCGCTGGCGCACGGCTT
CGCCCTGTTTGTGGCGGTGTCCGTGGGAGCTAACATCTCGGGTGGGCACG
TGAACCCGGCCGTGACCTTCGGCGCCTTCGTGGGCGGCAACATCACGCTG
CTACGAGGCATCCTCTACTGGATCGCCCAGCTGCTGGGCTCCACCGTGGC
CTGCCTCCTGCTCCGCTTCTCCACCGGCGGGCTCGAGACCGGCACCTTCG
GGCTGACCGGGGTGAGCGTGTGGGAGGCGCTCGTGCTGGAGATCGTCATG
ACCTTCGGCCTCGTGTACACCGTCTACGCCACCGCCGTGGACCCCAAGAA
GGGAAGTCTCGGCACCATCGCCCCCATCGCCATCGGCTTCATCGTCGGCG
CCAATATCCTTGTGGGCGGGCCCTTCGATGGCGCCTCCATGAACCCCGCC
GTGTCCTTCGGCCCGGCTCTCGTCAGCTGGTCGTGGACCCACCAGTGGGT
GTACTGGCTCGGACCACTCATCGGCGGCGCTCTTGCCGGTATCGTCTACG
AGATCTTCTTCATCAGCCACTCCCACGAGCAGCTCCCCACCGCCGATTAC
TGA

>MaTIP1-5
ATGCCGATCTCTCGGATCGCCATCGGGACGACGGAGGAGGCGACGCACCC
GAGCGCGCTGAAAGCCGCGCTCGCCGAGTTTATATGCACCTTCATCTTTG
TCTTCGCCGGCCAAGGCTCCGGCATGGCCTACAGCAAGATGACGAGCGGT
GGGGCGGCGACGCCCACAGGACTGATCATGGCGGCGCTGGCGCACGCCTT
CGCCCTGTTCGTGGCGGTGTCGGTGGGGGCGAACATCTCGGGCGGGCACG
TAAACCCGGCCGTGACCTTCGGTGCGTTCGTGGGCGGCAACATCACGCTG
CTGCGAGGCGTTCTCTACTGGGTGGCGCAGCTGCTCGGCTCCACCGCGGC
CTGCCTCCTGCTCCACTTCGCCACCGGCGGGCTGGAGACCGGCACCTTCG
GGCTGTCGTCGGGGGTGGGCGTGTGGGAGGCGCTGGTGCTGGAGGCCGTC
ATGACCTTCGGCCTTGTGTACACCGTCTACGCCACCGCCGTCGACCCCAG
GAGGGGTAGCCTCGGCGCCATCGCCCCCATCGCCATCGGCTTCATCGTTG
GCGCCAACATCCTGGTGGGCGGCGCCTTCGATGGTGCCTCCATGAACCCG
GCCGTGTCCTTCGGTCCCGCGCTCGTCAGCTGGTCGTGGACCCACCAGTG
GGTGTACTGGCTGGGCCCGCTCAGCGGTGGCGCCCTCGCCGGCCTCGTCT
ACGAGATCTTCTTCATCTGCAGCACCCACGAGCAGCTCGCCTCCGCCGAC
TACTGA

>MaNIP2-1
ATGGCTTCTTCCCATGTAAGGCCTAACAACTCCAACGAAATCCATGACAT
AGACGTCGTCACAGCTCAAACGTTGACCACCCCAAGCTTCTTCGACCCCC
CCAGAGTCCATCGTCGGAGAAATTTGAAGGAACTCTTTCCACCATTTCTT
CCCAGAAAGGTTGTGTCTGAGATGATAGCTACATTTTTACTTGTGTTCGT
GACCTGCGGAGCTGGTGCGTTAAACAAGAACAACCCGGGCGTGGTGTCGC
AATTAGGGCAATCGGTCGCCGGTGGCTTGATCGTCACTGTGATGATCTAT
GCGGTTGGCCATATTTCCGGGGCGCACATGAACCCCGCAGTCACGTTAGC
CTTCGCCGTGGCCCGGCATTTCCCATGGATACAGGTGCCCTTCTATATGT
TAGCTCAGATAGCAGGGTCTACGACCGCCTCGTACATCCTACGTGAGCTG
CTCGATCCCATTCATGATCTAGGGACGACGACGCCGTCTCACACTGCCGC
GAAAGCCTTGGTGGCGGAGATCGTAGTGACCTTTAACATGATGTTCGTGA
CCGCGGCGGTAGCGACGGACACAAAAGCCGTAGGAGAGTTAGCAGGCCTC
GCAGTTGGCTCAGCAGTTTGCATCACCTCCATCTTAGCGGGGCCCATCTC
GGGAGGGTCAATGAATCCAGCAAGGACGCTGGGACCGGCGTTGGCAAGCA
ACAAGTTTGACTCGCTGTGGGTGTACTTCGTTGGCCCGCCCGTGGGCACA
GTAGCAGGGGCTTTGGCCTACAGCTTTATACGACTAGATGAGCACTCGTT
ATCGTCACAGAAGGACAGCCAGAAGTCACCCTCCCTCAAGATGCGTCGCG
TGCAGAGCCAGGACATGGCGAGTCCCACTAATGATGCTTTTGAATCCGGC
GTTTAG

>MaPIP2-6
ATGTCGAAGGAGGTGAGCGTGGAGGTGGAGCAGCCACCGGCGAAGGACTA
CAGTGACCCGCCGCCGGCGCCGCTGCTGGACTTTGGTGAGGTCCGCCTCT

GGTCTTTCTACCGAGCCCTCATCGCCGAGTTCGTCGCCACCCTGCTCTTC
CTCTACGTCAGCATCGCCACCGTCATCGGCCACAAGGAGCAGAACGCGGC
CGACCAGTGCAGCGGCGTAGGCCTCCTCGGCATCGCTTGGGCCTTCGGCG
GCATGATCTTCATCCTCGTCTACTGCACCGCCGGAATCTCAGGAGGACAC
ATCAACCCGGCGGTGACGTTCGGGCTGTTCCTAGCGAGGAAGGTGTCGCT
GATAAGGGCGGTGCTGTATATTGTGGCTCAGTGCTTGGGAGGCATCGTCG
GGGTGGGCATCGTGAAAGGGATCATGAAACACCAGTACAACTCCCTCGGC
GGCGGGGCCAACGTGGTCGCAACCGGCTACTCCAAGGGCACCGCCCTCGG
GGCGGAGATCATCGGCACCTTCGTCCTCGTCTACACAGTCTTCTCCGCCA
CCGACCCCAAGCGCAGCGCCCGCGACTCTCACGTCCCCGTGTTGGCACCA
CTTCCCATCGGGTTCGCCGTGTTCATGGTACACCTCGCCACCATTCCCAT
CACCGGGACTGGCATCAACCCCGCTCGGAGCCTTGGCGCCGCAGTCATCT
ACAACCAGGACAAGGCCTGGGATGATCATTGGATCTTCTGGGTGGGTCCG
TTCATCGGAGCGTTGGCCGCCGCGGCGTACCACCAGTACATCCTGAGAGC
AGCCGCCATCAAGGCTCTGGGCTCCTTCAGGAGCAACCCGAGCAACTAA

>MaPIP1-2
ATGATCTTTGCCTTGGTCTACTGTACCGCCGGGATCTCTGGTGGCCACAT
CAACCCCGCGGTGACCTTCGGGCTGTTCCTGGCGAGGAAGCTGTCGCTGA
CCAGGGCTCTGTTCTACATGGTGATGCAGTGCCTGGGCGCCATCTGCGGT
GCCGGTGTGGTCAAGGGGTACCAGAAGGGGCTCTATGAGAGCAACGGTGG
CGGAGCTAACGTCGTGGCTCCTGGCTACACCAAGGGTGATGGCCTGGGTG
CTGAGATCGTCGGCACCTTCATCCTCGTCTACACTGTTTTCTCTGCTACT
GACGCCAAAAGAAACGCCAGGGACTCTCATGTTCCCATTTTGGCTCCCCT
CCCCATTGGGTTTGCCGTGTTCCTTGTCCACCTGGCTACCATCCCCATCA
CCGGCACCGGCATCAACCCTGCCAGAAGCCTTGGCGCTGCCATCATCTAC
AACAAGAAGCATGCCTGGGATGATCATTGGATCTTTTGGGTTGGACCCTT
CATTGGAGCTGCTCTTGCTGCCATCTACCACCAGATAGTCATCAGGGCGA
TCCCATTCAAGAGCAGGCCCTGA

>MaPIP2-11
ATGATCTTTATCCTCGTTTACTGCACCGCCGGCATCTCTGGTGGACACAT
AAACCCGGCCGTCACGTTGGGTCTGTTCCTGGCGCGGAAGGTGTCGCTGC
TTCGCGCGGTGATGTACATGGTGGCGCAGTGCTTGGGGGCCATATGCGGC
GTCGGGATCGTGAAGGGCATCATGAAGCACCAATTCAACGCCTTCGGCGG
CGGGGCAAACTCTGTAGCCGCAGGATACTCCAAAGGCACCGCGTTCGGCG
CCGAGAGCATCGGCACCTTCGTGCTCGTCTACACCGTCCTCTCCGCCACC
GACCCCAAGCGCAGCGCCCGCGACTCCCACGTCCCGGTGTTGGCTCCGCT
GCCCATTGGGTTCGCTGTGTTCATGGTTCACTTGGCCACCATTCCCATAA
CTGGCACGGGCATCAATCCGGCGAGGAGCTTCGGAGCCGCCGTCATTTAC
AACCAGCACAAACCCTGGCATGACCACTGGATCTTCTGGGTGGGTCCTTT
CGTCGGAGCTCTGGCGGCGGCGGTGTACCACCAGTACGTGCTGAGGGCGG

CGGCACTGAAAGCTTTGGGCTCGTTCAGGAGCAGCCGCAGCAACTAA

>MaPIP1-8
ATGATCTTTGCTCTGGTCTACTGCACTGCCGGCATCTCTGGTGGCCACAT
CAACCCGGCTGTGACCTTTGGGTTGCTACTGGCGAGGAAGCTCTCCCTGA
CCAGGGCCATCTTCTACGTGGTGATGCAGTGCCTGGGCGCGATTTGCGGC
GCCGGTGTGGTGAAGGGGTTTCAGAAGGGGGTGTACGAGAGCAACGGTGG
CGGAGCCAACGTCGTGGCCGCTGGCTACACCAAGGGCGATGGCCTTGGCG
CGGAGATCGTCGGCACCTTCATCCTTGTCTACACAGTCTTCTCCGCCACT
GATGCCAAGAGGAGTGCAAGGGACTCTCATGTGCCTGTGCTTGCACCCTT
GCCTATTGGATTTGCGGTTTTCCTGGTTCACCTGGCCACCATCCCCATCA
CTGGCACTGGCATCAACCCTGCCAGGAGCCTCGGAGCTGCCATCATCTAC
AACAAAGAACACGCCTGGAATGACCATTGGATCTTCTGGGTCGGTCCCCT
CATTGGAGCTGCCTTGGCTGCCATCTACCATCAGGTGGTGATCAGAGCCA
TCCCGTTCAGGAGCAAGCGTTGA

>MaTIP1-2
ATGCCGATCGGTAGCATAGCAATCGGAGCGCCCGGCGAGGCGAGCCATCC
CGACACGATCAAAGCCTCCCTCGCGGAGTTCATCTCCACGCTCATATTCG
TCTTCGCCGGCGAGGGCTCCGGGATGGCTTTCAATAAGCTAACAAATGAC
GGCTCCACGACGCCAGCAGGCCTGGTGGCGGCGTCCTTGGCCCACGGCTT
CGCCCTCTTCGTGGCGGTATCAGTCGGGGCTAACATCTCCGGTGGTCACG
TCAACCCGGCCGTCACCTTCGGGGCCTTCCTCGGCGGCAACATCTCTCTG
ATTCGCGGGATCCTCTACTGGATCGCGCAGCTGCTCGGATCCGTCGTCGC
CTGCCTGCTTCTCAAGCTCGCCACCGGTGGACTGGAGACGTCGGCCTTCT
CGCTGTCGTCGGACGTGAGCGTGTGGAACGCCGTGGTGTTCGAGATCGTC
ATGACCTTTGGGCTGGTGTACACGGTGTACGCGACGGCGGTGGATCCGAG
GAAGGGCGACCTGGGAGTCATCGCGCCCATCGCCATCGGGTTCATCGTCG
GCGCCAACATCTTGGCGGGCGGGGCGTTCGACGGCGCGTCCATGAACCCG
GCGGTCTCATTCGGCCCGGCGGTGGTCAGCTGGACCTGGGACAACCACTG
GGTGTACTGGGTCGGCCCGTTGATCGGGGCGGCCATCGCAGCTTTGGTCT
ACGACGGCGTCTTCATCGGCCAGGCGACCCACGAGCAGCTTCCCCCCTCA
GATTACTAG

>MaNIP4-1
ATGGTCTACTCTGTCGGCCACATCTCCGGTGCTCATTTTAATCCTGCGGT
CACCACCACGTTCACCATTCTCAAGCAGTTCCCTCTCAAGCAGCTGCCTC
TTTACATGGTAGCTCAGTTGGTAGGAGCCATCCTCGCAAGTGGGGCGGTG
TACTTGTTGTTCGATCCAAAAGCAGAGCACTTCTACGGAACTACGCCGGT
TGGATCAGCCGTGCAGTCCTTCGTCCTCGAGATCATCATATCTTTCCTCC
TGATGTTCGTCATCTCTGGCGTGGCCACAGACACCAGAGCTATTGGGGAA
TTAGCAGGCATTGCTGTCGGATCCACAATTTTGTTGAATGTCCTCGTTGC

CGGGCCGATATCAGGAGCGTCCATGAACCCTGCAAGAAGCATCGGACCCG
CCATCGTCATGCGTAACTACAAGGCAATCTGGGCCTACGTTTTAGGACCA
ATGATAGGTACTCTGGCTGGGGGTTTTACTTACAATCTCGTTAGATACAC
AGACAAGCCGCTCCGAGAGATCACAAAGAGCAGCTCATTCCTCAAGAGCG
TGTCTCGAAATCGTTAG

>MaSIP1-1
ATGGGTGCTATAAGGGCCGCCGCGGCGGACGGGTTGATCACGTTCCTATG
GGTCTTCTGCGTCTCTACCGTGCGCGCCGCCACCTCCCTCGTGACGGCCG
CCCTCCAGATCCAGGGCGTCGCCTTCTCGCTCTTTGTCACCACCACCCTC
ATCTTCGCCCTCGTCTTCGTCTTCGGCCTCATCACCGCCGCCATCGGCGG
CGCCAGCTTTAATCCCACTGCCACGGCCGCGTTCTATGCTGCCGGATTGG
GGTCCGACAACCTCCTCTCCATGGCGCTCCGCTTCCCCGCCCAGGCGGCT
GGGGCGGTGGCTGGGGCTTTGGCGATCATGGAAGTCATGCCTCCCCAGCA
CAAGCGCATGTTGGGCGGGCCTTCGTTGAAGGTGGATCTTCACACGGGGG
CCTTGGCGGAAGGTGTGCTGACCTTCATCATCACCTTGGCCGTGCTCTGG
ATCATCATCAGGGGGCCCTCGCAGCCCGGTCGTGAAGACGTGGATGGTCGC
CGTCTCCACGGTTGCGATGGTTGTAGCGGGCGCTGGTTACACTGGACCGG
CCATGAATCCAGCTAATGCATTTGGCTGGGCATATATCAACAATCGCCAT
AATACGTGGGAGCAATTTTACGTCTACTGGATATGCCCATTCATCGGCGC
TATTGTTGCTGGCTGGTTCTTCAGGATTATCTTCCCGCAGCGTGCAGAGA
AGGCTAAAAAAGCTTGA

>MaPIP2-8
ATGACGGACGAAGTAAGGGTAGTGACGGAGCACCCACCAGCGCCACTCTT
CGACGTGGGGGAGCTCAAGCTCTGGTCCTTCTATCGTGCTCTCATCGCTG
AGTTCGTTGCCACCCTCCTGTTCCTCTATGTCCTCGTGGCTACCGTCATC
GGCCACAAGGCCGCGTCTCTAGACAACCAGTGCGGCGGCGTCGGCCTCCT
TGGGATCGCTTGGGCTGTCGGCGGCATGATCTTTCTTCTCGTCTACTGCA
CCGCCGGCATCTCTGGTGGACACATCAACCCGGCCGTCACATTTGGTCTT
CTCCTAGGTCGAAAGGTGTCGGTGTTGCGGGCGGTGTCGTACATGGTGGC
GCAGTGTGCGGGCGCCATATGCGGCGTCGGAATCGCGAGGGCTATAATGA
AACACCAATTCGATGCTTTCGGCGGCGGGACTAACGTGGTGGCCTTATGC
TACTCCAACGGTGCTGCGCTTGGCGCTGAGATCATCGGCACCTTCGTGCT
CGTCTACACCGTCTTCACTGCCACCGACCCCACGCGCAACGCCCGTGACT
CCCACGTTCCGGTGTTGGCGCCTCTATCGATTGGCTTTGCGGTGTTTTTG
GTGCACTTGGCAACGATTCCGATCACAGGCACAGGTATCAACCCGGCAAG
GAGCCTCGGAGCTGCTGTCATTTACAACAGACGGAAGGCATGGAACGATC
AGTGGATCTTCTGGGTGGGTCCCTTCATCGGAGCTGCGGCCGCCGCGCTG
TACCACGAGTACGTGTTGAAGTCAGCGGCTATCAAGGCCTTCCGCAGGAG
CACCGCAACACCTGAAGCATCAGCTCCTCGTCCTCCTCCTCCGTGA

>MaTIP1-1

ATGCCGTTCTCTCAGATCGCCATTGGACGTCCGGAGGAGGCAACTCACCC
GAGTGCACTCAAGGCCGCGCTCGCTGAGTTCATATGCACCCTCATCTTCG
TCTTCGCCGGCCAAGGCTCCGGCATGGCCTACAACAAGTTGACGAGCGAC
GGGGCGGCGACGCCCGCGGGACTGATCGCGGCGGCGCTGGCGCACGGCTT
CGCCTTGTTCGTGGCGGTGTCGGTGGGGGCTAACATCTCCGGCGGGCACG
TGAACCCGGCGGTGACCTTCGGAGCGTTCGTGGGCGGGAACATCACGCTG
CTGCGGGGCATCCTCTACTGGATCGCGCAGCTGCTGGGCTCCACGGTGGC
CTGCCTCCTGCTCCGCTTCTCCACCGGCGGGCTCGAGACCGGCACCTTCG
GGCTGTCCGGGGTGAGCGCGTGGGAGGCGCTGGTGCTGGAGATCGTCATG
ACCTTCGGCCTCGTGTACACCGTCTACGCCACCGCCGTGGACCCCAAGAA
GGGCAGCCTCGGCACCATCGCCCCCATCGCCATCGGCTTCATCGTGGGCG
CCAACATCCTCGTGGGCGGGCCCTTCAGCGGCGCCTCCATGAACCCCGCC
GTGTCCTTCGGCCCGGCCCTCGTCAGCTGGTCCTGGACCCACCAGTGGAT
CTACTGGCTCGGTCCGCTCATCGGCGGCGGCCTCGCCGGGATCGTCTACG
AGTTCTTCTTCATCAGCCACTCCCACGAGCAGCTCCCCACCACCGACTAC
TGA

>MaPIP1-3

ATGATCTTTGCCTTGGTCTACTGCACCGCCGGCATCTCTGGTGGCCATAT
CAACCCTGCGGTGACCTTCGGGCTGTTCCTGGCGAGGAAGCTCTCCCTCA
CGAGGGCCATCTTCTACATGGTGATGCAGTGCCTGGGCGCCATCTGCGGC
GCCGGCGTGGTCAAGGGATTCCAAAAGGGTGTCTACGAGAACAACGGCGG
CGGAGCCAACGTCGTGGCCGCCGGCTACTCCAAGGGTGACGGCCTGGGCG
CCGAGATCGTGGGCACCTTCATCCTCGTCTACACCGTCTTCTCTGCAACC
GATGCCAAGCGTAACGCCAGGGACTCCCATGTCCCCATCCTTGCTCCATT
GCCCATCGGATTCGCCGTCTTCCTGGTTCACTTGGCGACCATCCCCATCA
CCGGCACCGGCATCAACCCCGCTCGGAGCCTGGGAGCTGCCATCATCTAC
AACAAGGACCACGCATGGGATGACCACTGGATCTTCTGGGTCGGTCCGTT
CATAGGAGCTGCCCTGGCCGCCTTCTACCACCAGATCGTCATCAGAGCCA
TCCCATTCAAGAGCAGATCCTGA

>MaPIP2-7

ATGTCGAAGGAGGTCAGTGAGGCCGAGCAGGCGCCGGCAAAGGACTACAG
GGACCCGCCGCCGGCGCCGCTTTTGGATTTCGGCGAGCTCCGTCTCTGGT
CCTTTTACCGCGCCCTCATAGCTGAGTTCGTGGCCACGCTGCTCTTCCTC
TACGTCACCATCGCCACCGTCATCGGCCACAAGGAGCAGAACGCCGCCGA
CCAGTGCAGCGGGGTTGGCATTCTTGGCATTGCGTGGGCCTTTGGTGGCA
TGATCTTCATCCTCGTCTACTGCACGGCCGGCATCTCTGGGGGACACATC
AACCCGGCGGTGACCTTCGGGCTGTTCCTGGCGAGGAAGGTGTCGCTGAT
ACGGGCGCTGCTGTACATAGTGGCGCAGTGCTTGGGAGCCATCGTTGGTG
TAGGGATCGTGAAGGGCATCATGAAGCACCAGTACAACTCTCTCGGTGGT

GGAGCCAACGAGGTCGCATCCGGCTACTCCAAGGGCACCGCCCTTGGAGC
CGAGATCATCGGCACCTTCGTCCTCGTCTACACCGTCTTTTCCGCCACCG
ACCCCAAGCGCAGCGCCCGCGACTCCCACGTTCCCGTGTTGGCACCACTC
CCCATCGGCTTTGCTGTGTTCATGGTGCACCTCGCCACCATCCCCATCAC
CGGTACCGGCATCAACCCCGCTCGGAGCTTAGGTGCTGCAGTGATCTACA
ACCAGGACAAGCCGTGGGATGATCATTGGATCTTCTGGGTGGGTCCGTTC
GTAGGAGCGTTGGCCGCGGCGGCGTACCACCAGTACATCCTGAGGGCAGC
GGCTATCAAGGCCCTGGGATCCTTCCGGAGCAACCCCACCAACTGA
